# Can Macromolecular Crowding Help Regulate Glutamate Dehydrogenase Activity?

**DOI:** 10.1021/acsomega.5c07618

**Published:** 2025-10-31

**Authors:** Genesis Rosario, Andrea Desrochers, Alec Robitaille, Emily Rundlett, Daniel Myšák, Zuzana Sochorová Vokáčová, Štěpán Timr, Eva Pluhařová, Kristin M. Slade

**Affiliations:** † 12295 Weill Cornell Medicine, Imaging, Midtown East 416 East 55th Street New York, New York, New York 10065, United States; ‡ 43317 Cornell University, College of Veterinary Medicine, 602 Tower Road Ithaca, New York, New York 14853, United States; § 640202 Corewell Health, Department of Dermatology, 18101 Oakwood Blvd, Dearborn, Michigan 48124, United States; ∥ 7712 University of Connecticut, School of Dental Medicine, 263 Farmington Ave., Farmington, Mansfield, Connecticut 06030, United States; ⊥ J. Heyrovský Institute of Physical Chemistry of the Czech Academy of Sciences, V. V. I., Dolejškova 2155/3, Prague 8 182 23, Czech Republic; # Department of Chemistry, Hobart and William Smith Colleges, New York 14456 300 Pulteney St, Geneva, New York 14456, United States

## Abstract

Glutamate dehydrogenase (GDH) is an important mitochondrial enzyme that is positioned at the intersection of several central metabolic pathways. Since this enzyme influences the flux of crucial metabolites, GDH activity is tightly controlled by a complex network of allosteric effectors, and disruption of this regulation has been correlated with a growing list of diseases. To better understand how the crowded environment and pH fluctuations of the mitochondrial matrix contribute to the fine-tuning of GDH regulation, Michaelis–Menten kinetics were measured in the presence of both synthetic and protein crowding agents. The results show a pH-dependent decrease in the GDH activity regardless of crowder identity. Specifically, macromolecular crowding favors the closed GDH conformation, thereby slowing product release. In addition, the presence of dextran increases the p*K*_a_ of a crucial lysine residue on GDH, while glucose, its small-molecule counterpart, does not. These kinetic results, together with Eyring plots and classical molecular dynamics simulations, suggest that excluded volume effects promote an abortive GDH-glutamate-NADH complex at lower pH (∼7). Under these conditions, crowding abrogates leucine activation but does not diminish GTP inhibition. Together, these findings indicate a complex interrelationship among macromolecular crowding, pH, and allosteric effectors to finely tune the GDH activity.

## Graphical abstract

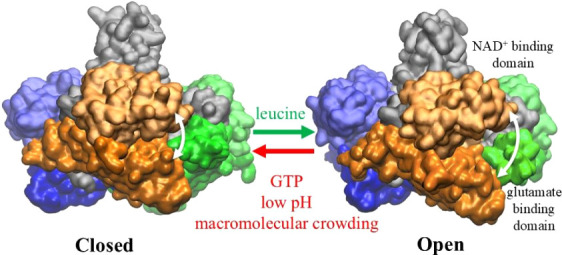

## Introduction

Glutamate dehydrogenase (GDH) catalyzes the reversible deamination of glutamate to alpha-ketoglutarate. Due to its crucial role in connecting nitrogen and carbon metabolism, GDH is heavily regulated by an array of metabolites using a complex, allosteric network. Dysregulation of this enzyme is linked to neurodegenerative disorders,^1^ hyperinsulinism,^2^ breast cancer,^3^ and other diseases.^4,5^

GDH regulation is intricately linked to its structure and mechanism. Most mammalian isoforms are homohexamers consisting of two trimers (Figure 1).^6^ Each GDH subunit contains a substrate-binding domain, a coenzyme-binding domain, and a regulatory domain. Mammalian GDH isoenzymes are unusual in their ability to use either NAD^+^ or NAD­(P)^+^ as a coenzyme,^7,8^ though evidence suggests that NAD^+^ is used exclusively *in vivo*.^9^ Random-order binding of glutamate and coenzyme to GDH initiates a conformational change, allowing one trimer to rotate past the other and close the catalytic cleft.^10,11^ This closure provides a hydrophobic environment and aligns the alpha-hydrogen of glutamate for hydride transfer to the NAD­(P)^+^ coenzyme.^12^ After catalysis, GDH, again, undergoes a conformational change back to its open conformation before products can be released. The two GDH trimers rotate away from one another as the catalytic cleft opens, expanding the entire core of the enzyme.^6^ These open and closed forms of GDH have distinct structures, exposing significantly different residues at the catalytic cleft.^13^ Evidence suggests that this closed-to-open transition, required for product release, is rate-limiting.^14^ Many allosteric effectors of GDH rely on this substantive conformational change to modulate enzyme activity.^15^ For example, the most well-studied inhibitor of GDH, guanosine triphosphate (GTP), promotes the closed conformation of this enzyme, preventing product release.^11^ In contrast, leucine, serving as a signal of protein abundance, activates GDH by favoring the open conformation and expediting coenzyme release.^16,17^

**Figure 1:**
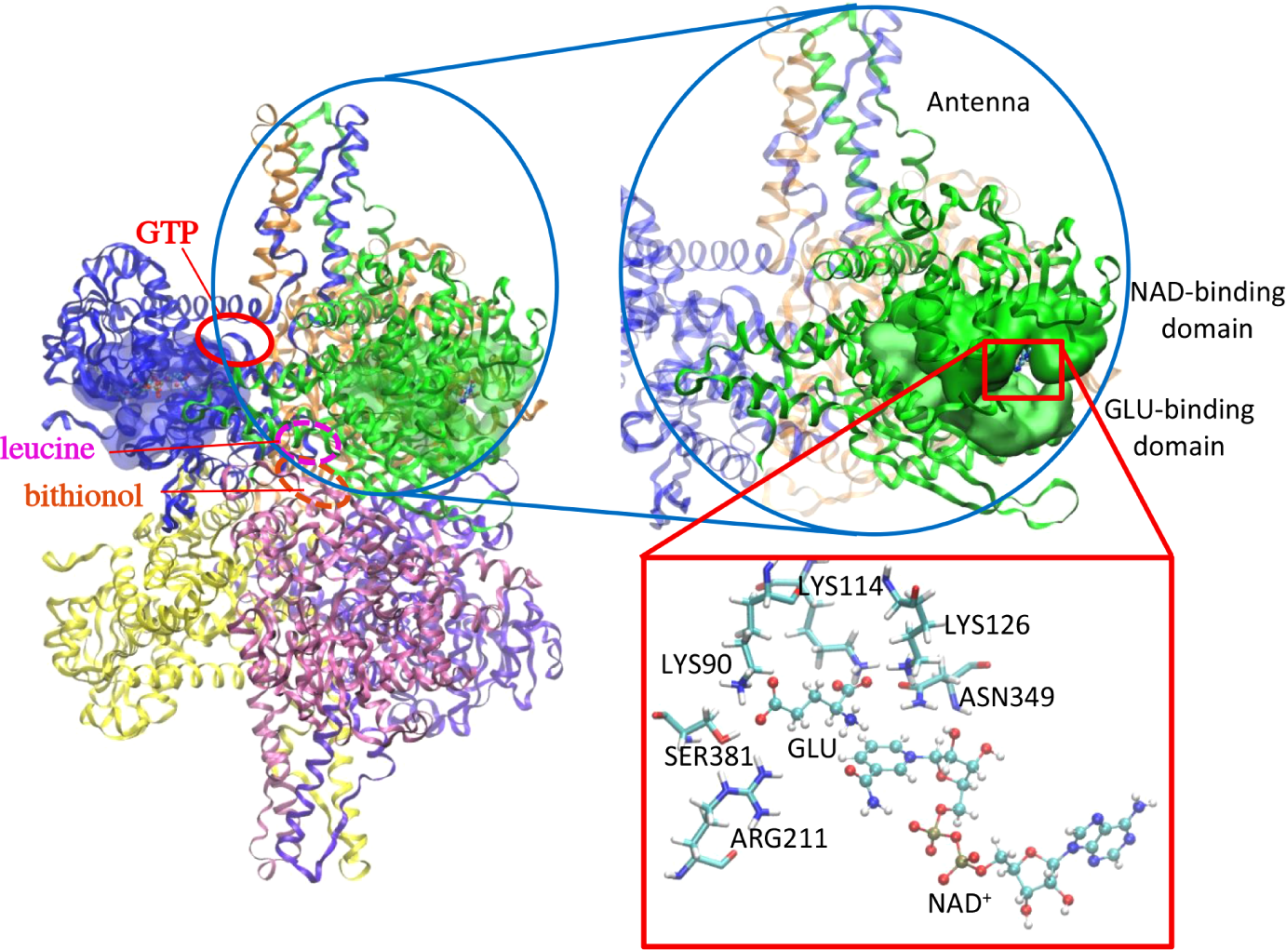
Structure of the bovine GDH hexamer (left) with a zoom-in on one of the six identical subunits (green). The antenna, the GLU-binding domain, and the NAD^+^-binding domain are labeled for the selected subunit. The red box shows the catalytic residues in the active site, the substrate glutamate (GLU), and the cofactor, NAD^+^. Ovals indicate the binding sites of GTP^11^ (red), leucine^17^ (pink), and bithionol^18^ (orange).

While its active site is highly conserved across species, only animal GDH exhibits allosteric regulation.^11^ GTP, ATP, NADH, and long-chain fatty acids, like palmitoyl CoA, serve as inhibitors, signaling an abundance of energy resources within the cell.^18^ In contrast, GDH is activated by both leucine and ADP. Together, the relative concentrations of these metabolites finely tune GDH activity to respond to the cell’s metabolic needs. Also unique to animal GDH is a 50-residue antenna region extending up from the NAD^+^-domain (Figure 1)^11^ that is likely to play a role in facilitating this allosteric regulation. The antenna is positioned just above the regulatory domain and undergoes a significant conformational change when the enzyme transitions between the open and closed states.^6^ This region facilitates communication between subunits of the enzyme and plays a major role in the allosteric regulation of GDH. Many GDH allosteric regulators, such as GTP, bind near this antenna in order to alter the ability of the enzyme to open the catalytic cleft as a means to modulate GDH activity.^13^ Point mutations to the antenna region desensitize the enzyme to GTP inhibition.^19^ This dysregulation of GDH results in excess insulin secretion and elevated levels of ammonia in patients with these genetic mutations.^4^

As evidenced by the range of diseases linked to GDH dysfunction, the GDH-catalyzed reaction sits at the crucial intersection of several important metabolic pathways. As such, the complex regulation of GDH is essential for fine-tuning glutamate flux and for maintaining the carefully balanced ratio of [NAD^+^]/[NADH]. Yet, recent studies are revealing that enzymes cannot be evaluated in isolation because they function differently in their native, crowded environment compared to the dilute solutions frequently employed for research.^20,21^ Cells consist of a heterogeneous mixture of proteins, DNA, carbohydrates, and other large molecules that can occupy up to 30% of their volume.^22^ Experimental results reveal that this crowding significantly influences enzyme kinetics by promoting binding, slowing diffusion, increasing effective concentrations, favoring oligomerization, and influencing conformational changes.^23^ In such densely packed environments, large molecules exclude volume from one another because they cannot occupy the same space.^24^ As a result, excluded volume effects decrease the entropy of the system, thereby increasing the thermodynamic activity. The system responds by shifting equilibria to favor more compact forms.^25^

Excluded volume effects are often studied by conducting experiments in the presence of high concentrations of large polymers like Ficoll, polyethylene glycol (PEG), and dextran,^21^ though some of these crowders are not as inert as originally anticipated.^26,27^ Weak interactions that are typically negligible in dilute solutions become significant at the high concentrations in crowded environments.^21,25^ Due to these soft interactions, protein crowders like bovine serum albumin (BSA), as well as mixtures of crowders or even cell lysate, are often preferred to synthetic polymeric crowders because they more accurately represent the internal environment of a cell.^26^ However, these protein-based models present additional challenges from a practical experimental design, often aggregating or interfering with the enzyme assay. To distinguish soft, enthalpic interactions from the entropically driven excluded volume effects, the small-molecule counterparts of these crowding agents can be employed.^28^ For example, glucose, as a small molecule, is unable to exclude volume to the extent of large crowders. At the same time, since dextran is a polymer of glucose, these two chemicals should share similar soft interactions.^29–31^ While protein crowders, mixtures of proteins, and cell lysates more accurately represent the true environment of a cell, studies with synthetic polymers, like dextran, are still essential to our understanding of macromolecular crowding because they provide more systematic control, allowing for the alteration of a single variable at a time, such as the size, concentration, or shape of the crowder. This information is crucial for building and verifying computational models of macromolecular crowding. In addition, the intracellular environment is incredibly complex. As advances in technology move experiments toward our ultimate goal of collecting data inside living cells, our insights from these artificial crowding studies will become even more valuable in helping to detangle which specific parameters have the most influence.^32,33^

While GDH has been well-studied, the majority of these experiments have been conducted in dilute (noncrowded) solutions at pH = 7; yet, the mitochondrial matrix can reach macromolecular concentrations upward of 560 g/L^34,35^ and its pH varies widely depending on cellular conditions.^36^ Many factors, such as calcium levels, energy state, and mitochondrial dysfunction, can alter this pH.^37^ Furthermore, the matrix pH plays a major role in metabolic regulation, and fluctuations can even trigger cellular apoptosis.^38^ It is possible that cells use variation in the matrix pH as one of many factors used to regulate GDH activity. Several recent studies have explored the effects of both pH and macromolecular crowding as separate, parallel factors impacting biological systems.^39–42^ However, these studies do not investigate the connection between these two physicochemical parameters or how they influence one another’s effects. As such, exploring the connection between pH and crowding effects is clearly an understudied area worthy of pursuit.

The GDH mechanism is heavily influenced by pH, mainly due to three essential lysine residues, which have abnormally low p*K*_a_ values around 8.^14^ First, lysine 126 (Figure 1) must be deprotonated before the catalytic cleft of GDH can close, a necessary step preceding catalysis.^43^ However, in lower pH solutions, when lysine 90 and 114 of the substrate-binding pocket are protonated, GDH has a higher affinity for glutamate. This higher affinity is problematic because GDH is prone to forming tightly closed, abortive complexes with glutamate and NADH. The presence of the abortive complex manifests as substrate inhibition at high glutamate concentrations in the kinetic data. This phenomenon becomes less prevalent as the pH of the solution increases and the enzyme’s affinity for glutamate decreases. As a result, the GDH reaction is faster at higher pH values.^43^

The goal of this work is to examine the joint effects of pH and macromolecular crowding on the steady-state kinetics of GDH in an effort to shed light on its regulation. Combining Michaelis–Menten assays with molecular dynamics simulations, we show that in a crowded environment GDH activity decreases and that the enzyme is more responsive to GTP inhibition. These findings provide insight into the pH-dependent role of macromolecular crowding in the complex, allosteric regulation of GDH.

## Results

Macromolecular crowding decreases GDH activity in a concentration-dependent manner, with the relative *V*_max_ value decreasing as dextran, BSA, or glucose concentrations are increased (Figure 2A). Unlike other large enzymes,^44^ GDH activity is independent of the crowder size or identity (Figure 2B). Similar crowding effects were observed regardless of whether the coenzyme was NADP^+^ or NAD^+^ (Figure 2C). Crowding also decreased the Michaelis–Menten constant of GDH (Figure S1). This decrease in the K_m_ of glutamate is mainly independent of the concentration and size of the crowder. In general, dextran has more of an effect on the K_m_ of glutamate than on the K_m_ of either coenzyme, NAD^+^ or NADP^+^ (Figure S2C).

**Figure 2:**
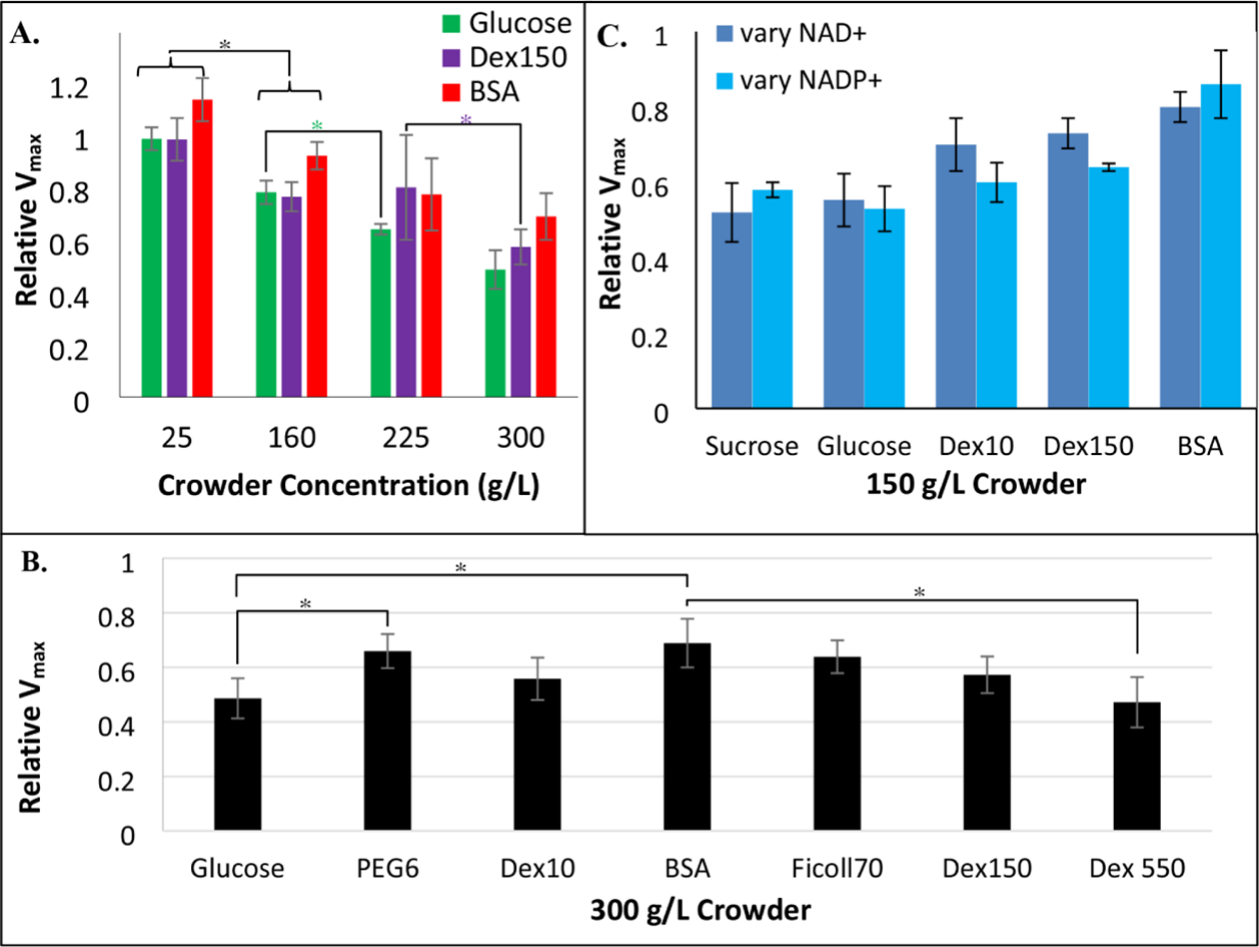
Crowding decreases glutamate dehydrogenase activity. 60 nM GDH assays in 100 mM phosphate buffer (pH = 7.0) with (A,B) 1 mM NAD^+^ and varying glutamate concentrations, or (C) 20 mM glutamate at varied NAD^+^ or NADP^+^ concentrations were performed in the presence of (A) varying concentrations, (B) 300 g/L, or (C) 150 g/L crowder. *V*_max_ values from the resulting Michaelis–Menten curves were normalized to values acquired in buffer only to yield “relative *V*_max_” values. Error bars represent standard deviations (*n* = 3). (A,B) Statistical differences in relative *V*_max_ values are indicated by asterisks (*p < 0.05, two-tailed). (A) All crowders showed a statistical difference (*p* < 0.05) when employed at 25 vs 160 g/L, as indicated by the bracketed bars. (C) Student’s two-tailed *t*-test failed to show any statistical difference between relative *V*_max_ values with NAD^+^ vs NADP^+^.

To better simulate the heterogeneous nature of a cell, the GDH assay was exposed to a binary mixture of BSA and dextran, as well as egg white, which contains over 40 different proteins.^45^ The presence of 100 g/L egg white decreases the glutamate K_m_ to relative K_m_ = 0.16 but has little effect on the *V*_max_ (relative *V*_max_ = 0.99). In contrast, the presence of a 1:1 mixture of BSA and dextran decreases both *V*_max_ and K_m_ (Figure S2). None of the crowders alter the stability of GDH based on the guanidinium and temperature denaturation curves (Figure S3).

Since excluded volume promotes associations and binding, a possible mechanism by which crowders could be decreasing GDH activity is by promoting the formation of the glutamate•GDH•NADH abortive complex observed at high glutamate concentrations.^46^ To investigate this possibility, the GDH assay was repeated for a larger range of glutamate concentrations, and the data was fit to a modified Michaelis–Menten equation with an inhibition constant, K_i_, to account for substrate inhibition (see section Methods). At pH 7, the presence of PEG (Figure S4), dextran, or BSA (Figure 3A) decreased K_i_, indicating an increase in the level of substrate inhibition. As the pH of the solution was increased, the measured K_i_ value increased regardless of the presence of a crowder. The pH dependence with BSA was unable to be investigated because solutions with pH values above 8 caused the BSA protein to aggregate. While Figure 3A suggests that glucose may also enhance substrate inhibition, the measured K_i_ in glucose was not statistically different than the K_i_ in buffer (Figure 3B).

**Figure 3:**
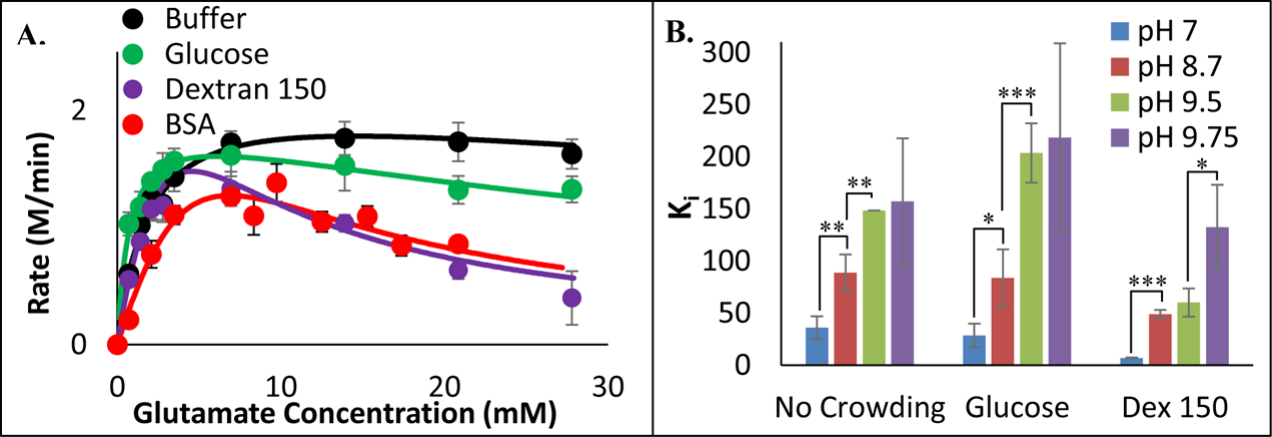
GDH substrate inhibition is pH-dependent. (A) Assays containing 1 mM NAD^+^, 150 nM GDH, and varying glutamate concentrations were run at pH = 7.0 in 100 mM phosphate buffer (black), 200 g/L glucose (green), dextran 150 kDa (purple), or BSA (red). The resulting data was fit to the Michaelis–Menten equation modified for substrate inhibition (eq 2). (B) GDH assays were run at pH 7 (blue), 8.7 (maroon), 9.5 (green), and 9.75 (purple), except that pyrophosphate buffer was employed. and the resulting inhibition constants K_i_ values were obtained from best fits of eq 2 Error bars represent standard deviations (*n* = 3). Asterisks (*p < 0.1; ** *p* < 0.03; *** *p* < 0.01, two-tailed) indicate a significant difference in K_i_ values as indicated.

Given the substantial influence of pH on GDH kinetics, we sought to investigate if the crowding effects also varied with pH. Dextran has a greater influence on GDH activity at lower pH values, whereas the effects from glucose were less pH-dependent (Figure S5). The resulting sigmoidal curves (Figure 4) were fit to eq 3 (see section Methods) to obtain the p*K*_a_ value of a crucial residue necessary for GDH catalysis. Interestingly, this p*K*_a_ value increased in the presence of dextran compared to buffer alone (*p*-value = 0.05, two-tailed *t*-test), but not in the presence of glucose (Table 1).

**Figure 4:**
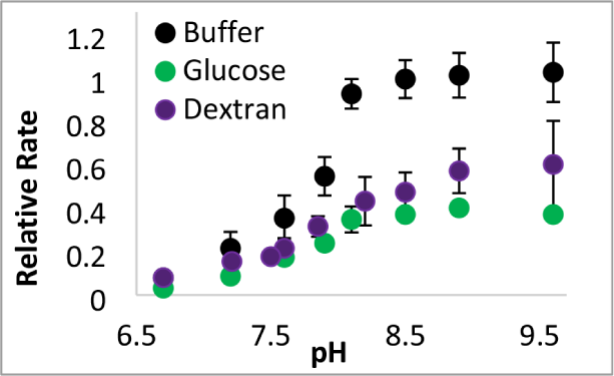
pH-dependence of crowding effects. GDH kinetic assays were run in varying pH buffers in the absence (black) or presence of 300 g/L dextran 150 (purple) or 300 g/L glucose (green). Each assay contained 9 mM glutamate, 1 mM NAD^+^ and 150 nM GDH added last to initiate the reaction. All rates were divided by the highest rate in buffer only to obtain the relative rates. Error bars represent standard deviations (*n* = 3).

**Table 1:** Dextran Alters the p*K*_a_ of a Crucial Residue Involved in GDH Catalysis

	p*K*_a_ ^a^
Buffer	7.8 ± 0.3
Glucose	7.7 ± 0.4
Dextran 150	8.4 ± 0.2

a Values determined from initial GDH rates as a function of pH (Figure 4).

When norvaline was used as an alternative substrate, the presence of glucose enhanced the rate of GDH activity in pH solutions below the crucial p*K*_a_ value, while the effects from dextran were similar for both substrates (Figure S5). Norvaline data was unable to be collected at lower pH values due to the extremely low GDH activity.^47^ The affinity of GDH for norvaline decreases significantly at low pH values.^48^

To further understand the influence of the crowding effects on the enzyme mechanism, we altered the order of adding reagents to the assay. Rather than adding enzyme last, GDH was mixed with glutamate and allowed to equilibrate for 10 min before adding NAD^+^ to start the reaction. In both the presence and absence of glucose, this premixing of glutamate and GDH slowed the reaction to about 60% of the rate of the standard assay, regardless of pH (Figure 5). The extent to which premixing impeded the GDH activity in the presence of Dextran was pH-dependent, having more of an effect at lower pH values. In contrast, premixing NAD^+^ with GDH had no effect on the reaction rate compared to the standard assay of adding enzyme last (Figure S6 orange squares). When norvaline was used as the substrate, the premixing results were within error for the standard assay rates (Figure S7).

**Figure 5:**
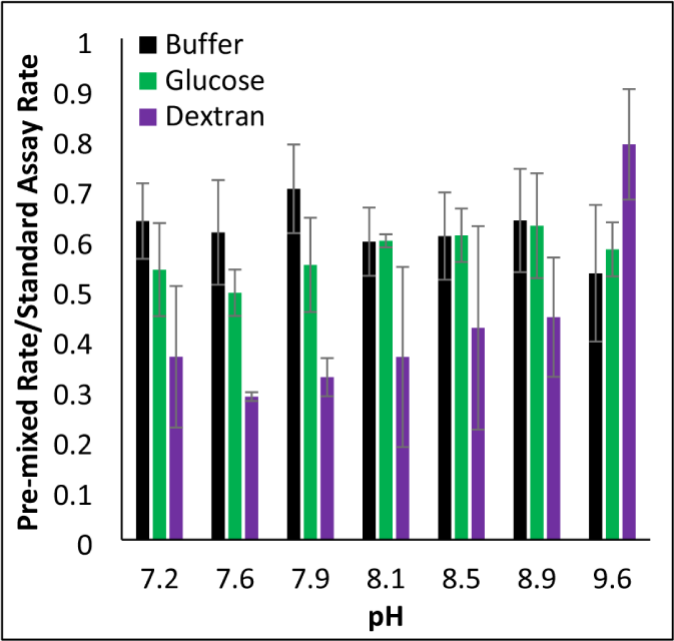
Premixing of GDH with glutamate. GDH kinetic assays were run at varying pH in the absence (black) or presence of 300 g/L of dextran 150 (purple) or 300 g/L of glucose (green). Each assay contained 150 nM GDH, 9 mM glutamate, and 1 mM NAD^+^, but the order of addition differed. In the standard assay, GDH was added last to initiate the reaction, or GDH was premixed with glutamate for 10 min before adding NAD^+^ to initiate the reaction. Premixed rates were divided by the corresponding rate from the standard assay. Error bars represent standard deviations (*n* = 3).

Crowding effects are often categorized as soft interactions or excluded volume.^30^ To begin to understand the source of the crowding effects on GDH steady-state kinetics, turnover rates, *k*_cat_, were collected as a function of temperature at saturating concentrations of glutamate and NAD^+^ (Figure S8). The resulting data was fit to the Eyring equation (eq 4, see section Methods) to determine the enthalpy (ΔH^⧧^) and entropy (ΔS^⧧^) of activation values (Table 2). At pH 8.5, the thermodynamic parameters were within error of one another, regardless of whether a crowder was present. In contrast, at pH 7, the presence of large crowders increased ΔH^⧧^ and ΔS^⧧^ (lower magnitude negative value, peach highlighting in Table 2) compared to the dilute solution, while glucose did not alter these parameters. In general, increasing the temperature from 25 to 37 °C had little influence on the kinetic effects of glucose or dextran (Table S1).

**Table 2:** Thermodynamic Activation Parameters for GDH^a^

	**Buffer**		**300**g/L **Glucose**		**300** g/L **Dex150**		**300** g/L **BSA**	
	**p**H = 7	**p**H = 8.5	**p**H = 7	**p**H = 8.5	**p**H = 7	**p**H = 8.5	**p**H = 7	**p**H = 8.5
**ΔH^#^ kJ/mol**	35 ± 4	20 ± 2	33 ± 2	23 ± 7	50 ± 15	22 ± 3	55 ± 8	22 ± 10
**ΔS^#^ J/mol K**	**–170 ± 20**	**–170 ± 10**	**–170 ± 40**	**–160 ± 20**	**–130 ± 50**	**–170 ± 10**	**–150 ± 10**	**–179 ± 15**

a Values determined from Eyring plots (Figure S8).

In order to determine if enhanced substrate inhibition is the main mechanism by which crowding slows GDH activity, the assay was exposed to leucine, which is known to alleviate substrate inhibition by opening the GDH complex.^49^ At pH 7, the presence of leucine increases the substrate inhibition constant, K_i_, in buffer alone but has little influence on the K_i_ in the presence of dextran or glucose (Figure 6A). In contrast, at pH values above the critical p*K*_a_ of GDH (Table 1), the presence of leucine significantly diminishes the effects of both glucose and dextran (Figure 6B). At pH = 9, leucine has no effect on the K_m_ of glutamate (Table S2), but it substantially increases the *V*_max_ (Figure S9A). In opposition to leucine, GTP serves as an inhibitor of GDH by stabilizing the closed conformation. Thus, increasing concentrations of GTP slow the rate of GDH in buffer (Figure 7) and decrease the K_m_ of glutamate (Figure S9 and Table S2).

**Figure 6:**
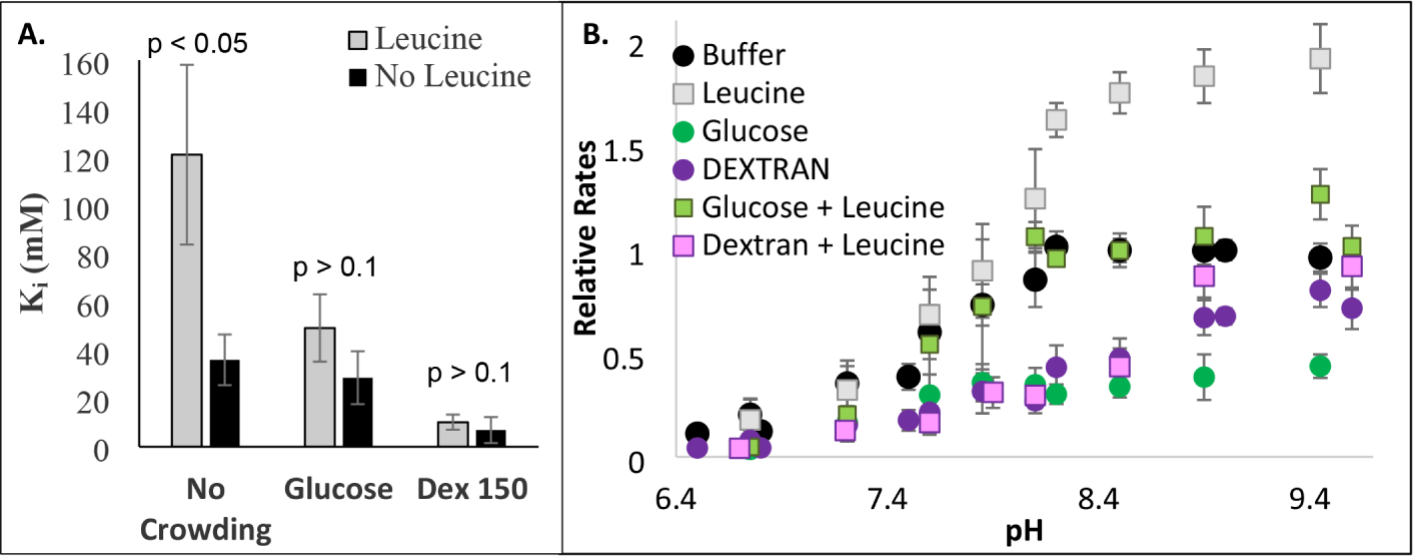
Effects of crowding on leucine activation of GDH. Kinetic assays with (squares) and without (circles) 10 μM leucine were run with 1 mM NAD^+^, 60 nM GDH, 9 mM glutamate with 300 g/L glucose or dextran 150 in (A) 0.1 M phosphate buffer at a pH of 7.0 or (B) 0.1 M pyrophosphate buffer with 10 μM EDTA. Error bars represent standard deviations (*n* = 3). To compare the K_i_ values with and without leucine, *p*-values (two-tailed) were calculated for *t*-tests, assuming equal variance (A).

**Figure 7:**
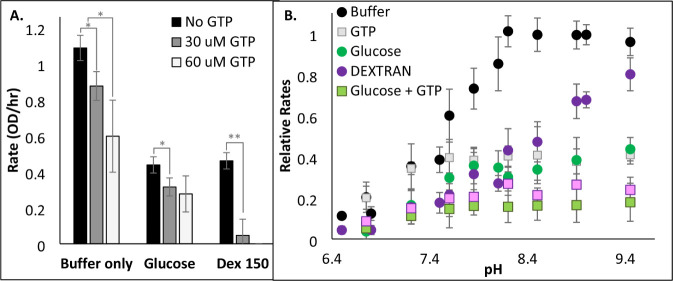
Effects of crowding on GTP inhibition of GDH. GDH kinetic assays were run with 1 mM NAD^+^, 60 nM GDH, 9 mM glutamate with 300 g/L glucose or dextran 150 in (A) 0.1 M phosphate buffer at a pH of 7.0 or (B) 0.1 M pyrophosphate buffer with 120 μM GTP. Error bars represent standard deviations (*n* = 3). Asterisks (*p < 0.05; ** *p* < 0.01, two-tailed) indicate a significant difference in rates with or without GTP (at either 30 or 60 μM GTP as indicated).

To begin to investigate the impact of crowding on potential drug candidates, the rate of the GDH assay was exposed to known GDH inhibitors, bithionol^15^ and zinc,^47^ in the presence and absence of dextran and glucose (Figure S10). In solutions containing dextran, bithionol had no additional inhibitory effect on the GDH activity. Contrary to literature reports, zinc had little effect on the GDH activity under the conditions used in this assay.^47^

Classical molecular dynamics (MD) simulations with empirical force fields (FF) can bring molecular-level insight into the GDH structure and dynamics as well as into the interactions of the substrates with the active site residues. The interplay between the open and closed conformations plays an important role in the reaction mechanism. Figure 8A shows the angle between the NAD^+^ and glutamate-binding domain (see section Methods for definition). This angle was monitored for each subunit of the simulated trimer as a function of the simulation time in the presence or absence of substrates—glucose, dextran, and GTP—at pH 7 (Figure 8B) or 9 (Figure 9). The apoenzyme (Figure 8B, 1^st^ row) can adopt a widely open arrangement, as indicated by large angles. The closed-to-open conformational change of subunit B in buffer (orange) is illustrated in Figure 8A. Interestingly, on our simulation time scales (hundreds of nanoseconds), individual subunits behave independently and can open and close again. Addition of the reactants, glutamate and NAD^+^, to the enzyme (Figure 8B, 2^nd^ row) brings the domains closer together. Such an effect seems to be the most pronounced in the presence of dextran. The addition of GTP promotes an even more closed conformation. Finally, the replacement of glutamate by norvaline at pH 7 leads to an opening. However, the increase of pH to 9 (Figure 9) in the presence of norvaline reduces the angle again, probably because norvaline fits better in the less polar active site, as will be described later.

**Figure 8:**
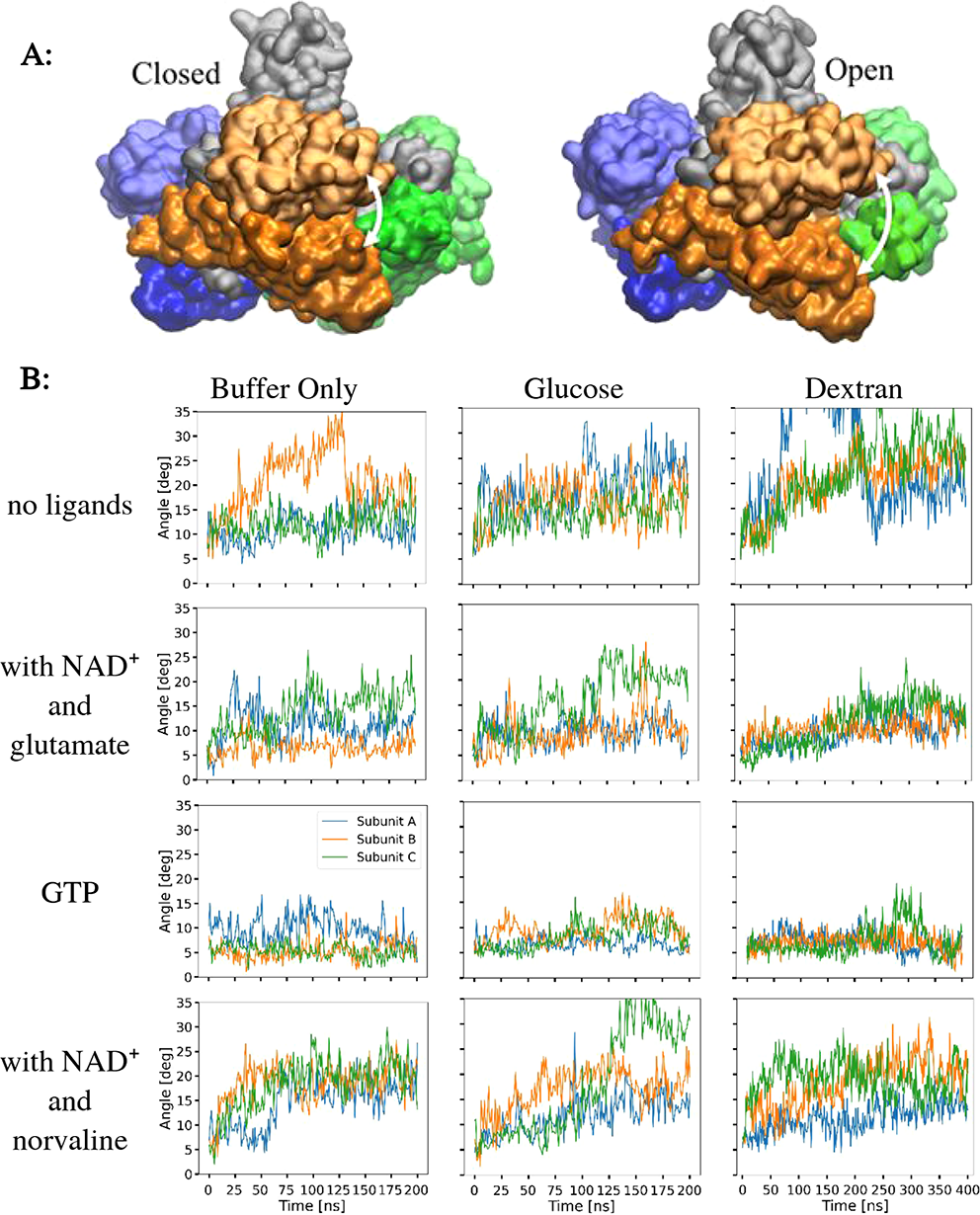
Time evolution of the angle between the NAD and GLU domains at pH 7. (A) Closed and open conformations of bovine GDH with subunit A (blue), subunit B (orange), and subunit C (green). The glutamate domain is glossy. The NAD^+^ domain is matte. (B) Time evolution of the angle between the NAD and GLU domains (white arrow in part A) calculated for the glutamate dehydrogenase trimer complex. In rows: 1. GDH without substrates; 2. GDH with NAD^+^ and GLU; 3. GDH with NADPH, GTP, and GLU; 4. GDH with NAD^+^ and norvaline.

**Figure 9:**
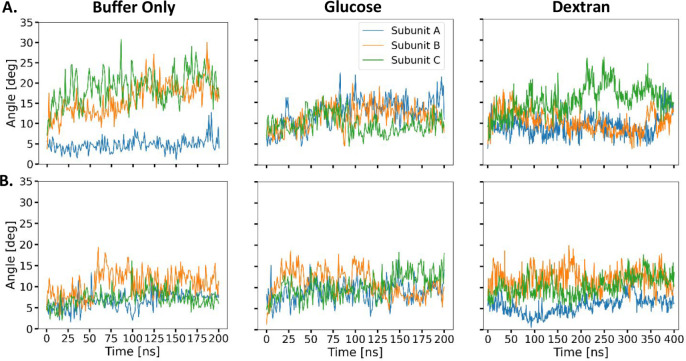
Time evolution of the angle between the NAD and GLU domains at pH 9. Calculated for the glutamate dehydrogenase trimer complex with NAD^+^ and (A) glutamate or (B) norvaline in buffer only, 100 g/L glucose, or 100 g/L dextran. Colors correspond to different subunits (A: blue, B: orange, C: green).

The angle between the domains probes one type of protein motion. The overall structural flexibility is commonly characterized by root-mean-square fluctuations (RMSF, Figure S11). While similar among the different systems, the major differences are fluctuations in the RMSF values for the residue region between 200 and 300, which corresponds to the catalytic mouth.^5^ The highest values in this region were obtained for the system containing norvaline and NAD^+^ in a glucose solution. Higher RMSF values correlate with the larger interdomain angle (Figure 8), but the correlation is not absolute because the protein domains are flexible objects. If they change shape, the principal axis of the fitted ellipsoid might slightly change the direction too. Finally, the values of the root-mean-square deviations (RMSD) from the reference crystal structure (Figure S12), together with the visual inspection in VMD, suggest no dramatic structural rearrangements. Higher RMSD values correlate again with a larger angle (Figure 8).

Our MD simulations also provide insight about the pH-dependent binding trends of each GDH substrate observed in previous experiments.^43^ At pH 7, the carboxylic groups of glutamate interact with positively charged Lys and Arg side chains (Figure 10A). Even though glutamate maintains most of the depicted attractive interactions that closely resemble the crystal structure, it has some flexibility (Figure S13A). The water molecules from the surrounding solution could enter into the active site. Arg211 sometimes undergoes a conformational change and forms a salt bridge with Glu173 (Figure S13A orange circle). At pH 9, glutamate adopts a different conformation in the active site. Specifically, the deprotonation of the three crucial lysine residues leads instead to the formation of salt bridges between the α-COO^–^ of glutamate and Arg211. These lysine residues, now neutral −NH_2_, accept hydrogen bonds from the −NH_3_^+^ group of glutamate (Figure 10A, pH = 9). Alternatively, at pH 9, both carboxylic groups of glutamate can interact with different parts of Arg211 or with the α-COO^–^ group with Arg94 (Figure S13B). Such arrangements are not suitable for chemical transformation and are less stable than the arrangements at pH 7. In one case, at pH 9, glutamate even resulted in spontaneously leaving (Figure S15A), but this was never observed at pH 7.

**Figure 10:**
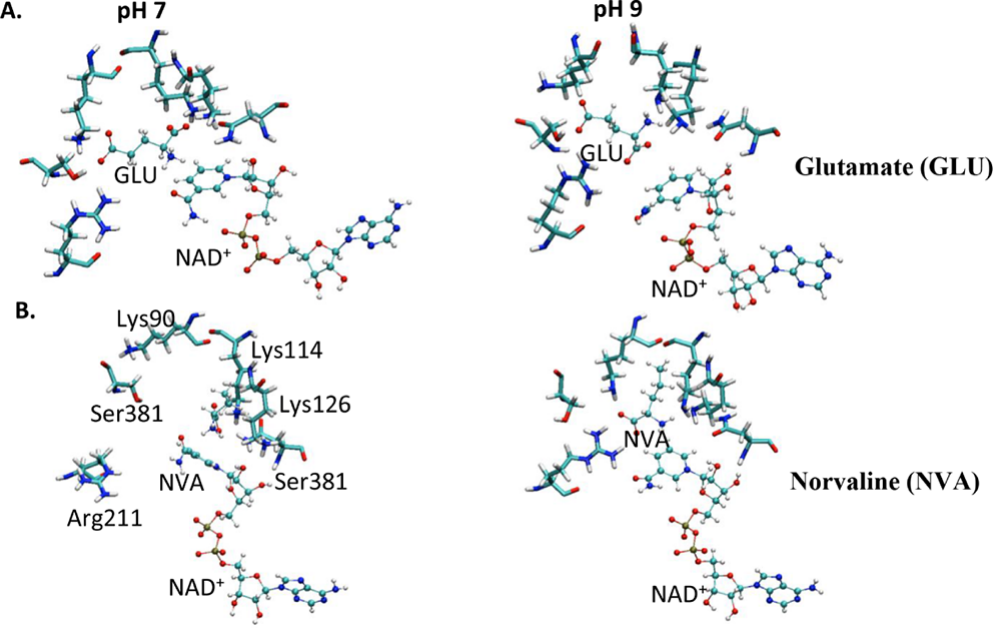
pH-dependence of substrate arrangement in the active site of GDH. Molecular dynamics simulations were performed for (A) glutamate (GLU) or (B) norvaline (NVA) at pH 7 (left) or pH 9 (right).

In comparison with glutamate, norvaline, possessing a −CH_3_ group instead of γ-COO^–^, is less polar and thus weakly binds the GDH active site at pH 7. This poor fit is exemplified by Arg211 moving away from the nonpolar part of norvaline (Figure 10B, pH = 7) and instead forming a salt bridge with Glu173 (Figure S14A). The nonpolar part of norvaline interacts with the nonpolar parts of the active site residues or even triggers the nonpolar side chains of Val378, Met111, Ala166, or Pro167 (Figure S14A) close to it. At pH 7, norvaline can spontaneously leave the active site, but in the opposite direction as glutamate (Figures S15B and S16). In contrast, the less polar active site at pH 9 is a better fit for norvaline (Figure 10B, pH = 9). The hydrophobic region of this substrate is aligned with the nonpolar part of the neutral Lys side chains and can interact with Met111 (Figure S14B). Additionally, the COO^–^ of norvaline is oriented toward Arg211, and the −NH_3_^+^ donates hydrogen bonds to the −NH_2_ of the Lys residues, which altogether result in a compact arrangement.

The components interact with the surface of the protein and can enter into the region between the domains. Water, as a small solvent molecule, can penetrate deep into the active site, even between the amino acid side chains and glutamate (Figure S13A). Glucose, which is larger, cannot enter as far. Our molecular dynamics simulations show that this sugar frequently interacts with NAD^+^. Once, we observed the formation of a hydrogen bond between glucose and glutamate γ-COO^–^ (Figure S17B, orange circle). The dextran polymers can be present in the interdomain region and can interact with the cofactor too, but less frequently than glucose. This difference is probably due to the size of the dextran polymer and the need to adopt a particular conformation to fit.

## Discussion

Given the complexity of GDH regulation and its importance in maintaining proper metabolic flux, we sought to investigate whether macromolecular crowding and pH might together play a combined role in this process. Indeed, the data reveal that the complex mechanism by which crowding impedes GDH activity is pH-dependent. First, crowding decreases *V*_max_ in a concentration-dependent manner that is independent of the crowder identity (Figure 2A), suggesting that this decrease likely results from excluded volume effects. Macromolecular crowding theory reveals that excluded volume effects should shift equilibria to maximize entropy and available volume, thereby favoring the most compact protein conformation.^50^ As such, we hypothesize that the presence of BSA, dextran, or PEG should favor the closed GDH conformation, which would impede product release and slow the enzyme activity. Our molecular dynamic simulations confirm that the presence of dextran with NAD^+^ and glutamate results in smaller angles between GDH subunits (Figure 8) and lower RMSD values (Figure S12), indicative of the closed conformation. In contrast, the subunit angle and RMSD values for GDH with NAD^+^ and glutamate were the same regardless of whether glucose was included in the simulation. This theory is further supported by the fact that crowding similarly impedes GDH activity when NAD^+^ is replaced with NADP^+^ (Figure 2C). If crowding instead altered the catalytic step, then one would expect the relative *V*_max_ values to vary with different substrates. In contrast, the excluded volume should have the same effect on the GDH conformational change required for product release regardless of the substrate.

Second, these crowders appear to enhance substrate inhibition by promoting the formation of the glutamate•GDH•NADH abortive complex (Figure 3). This form of GDH has a tightly closed NAD^+^-binding domain^11^ and should consequently be favored by excluded volume effects over the open conformation. Furthermore, GDH substrate inhibition is pH-dependent (Figure 3) because glutamate binds to GDH more effectively at low pH when lysine 114 and 90 are protonated.^14^ As pH is increased, the binding affinity of GDH for glutamate decreases, and thus less substrate inhibition is observed. This weaker binding is supported by the less favorable glutamate accommodations in the active site at pH 9 (Figure 10A) and the ability for glutamate to spontaneously leave (Figure S15). The enhanced glutamate substrate inhibition from dextran at low pH values explains the deviation in crowding effects for the alternative substrate norvaline at the lowest measurable pH value (Figure S5, orange vs purple data at pH = 7.8). Norvaline is unable to form any abortive GDH complex, and thus no substrate inhibition is observed.^47,48,51^ Dextran uniformly impedes product release of GDH regardless of substrate and thus slows the reaction, but at low pH values, the glutamate reaction is further impeded by substrate inhibition, while the norvaline reaction is not.

An enhancement in substrate inhibition from crowding at a low pH is consistent with the measured thermodynamic parameters. At pH 7, the addition of large crowders (dextran or BSA) increases both the activation entropy, ΔS^⧧^ and enthalpy, ΔH^⧧^ (Table 2, peach-highlighted data) compared to glucose or dilute solutions, but no difference in these thermodynamic parameters is observed at pH 8.5. This enthalpy–entropy compensation in which ΔG^⧧^ remains relatively unchanged was previously observed when comparing psychrophilic and mesophilic enzymes.^52^ The increased ΔH^⧧^ with mesophilic enzymes was attributed to a less flexible exterior of the enzyme, which causes a decrease in the free energy landscape compared to the wider range of conformational samplings for psychrophilic enzymes. This claim is supported by Figure S11 (second row). With NAD^+^ and glutamate at pH 7, the presence of dextran seems to lower the RMSF in GDH residues 250–300, suggesting less flexibility. Similarly, the presence of dextran or BSA is likely to restrict the flexibility of the exterior mobile surfaces of GDH. This decreased flexibility means less of an entropy cost when the substrate is converted to the transition state.

To confirm the theory that macromolecular crowding promotes the closed conformation of GDH, the enzyme assay was exposed to dextran in the presence of a GDH activator or inhibitor. Specifically, the small-molecule inhibitor, bithionol, binds to hydrophobic residues at the core of the GDH dimer interface.^15^ If crowders such as dextran impede GDH activity by the same mechanism as bithionol, then no additional inhibition should be observed when bithionol is added to the dextran assay, as observed in Figure S10. The same results were anticipated with GTP, which also inhibits GDH by promoting the closed conformation. Instead, additional inhibition was observed when dextran and GTP were both added to the GDH assay compared with only one of these inhibitors (Figure 7). This observation suggests that the crowder further stabilizes the closed GDH conformation beyond that which GTP can do on its own. The molecular dynamics simulations with GTP and dextran also support this claim (Figures 8 and S12).

The disparate results with bithionol and GTP in the presence of dextran may be related to where each inhibitor binds (Figure 1). While both stabilize the closed conformation, GTP binds just below the antenna region of GDH, whereas bithionol binds at the subunit interface near the GDH core, serving as a wedge to prevent cleft opening.^6^ It is possible that the excluded volume effects from dextran stabilize the closed GDH conformation in a mechanism similar to that of bithionol that does not involve the antenna required by GTP. To test this hypothesis, kinetic assays were run in the presence of dextran and leucine, an activator that binds at the GDH dimer interface, similar to bithionol. Leucine facilitates the transition to the open conformation after catalysis, thereby improving the product release and preventing substrate inhibition. GTP inhibition requires this antenna region, whereas leucine and bithionol do not. Unlike GTP, they maintain their potency even with GDH mutants lacking the antenna region.^11^ The results suggest that both dextran and glucose abolish leucine activation but only at low pH 7 values (Figure 6). In fact, below pH 8.4, the p*K*_a_ of the crucial residue for GDH, leucine, has no ability to alter the effects with dextran (Figure 6B: compare purple and pink data). Furthermore, dextran was still able to promote substrate inhibition in the presence of leucine, while glucose could not (Figure 6A black bars). At pH 9, when the abortive complex is no longer prevalent, the effects of dextran and leucine seem to partially counteract one another (Figure S9A). Taken together, the results with crowding and the allosteric inhibitors suggest that crowding promotes the closed conformation of GDH by influencing the dimer interface, not the antenna region.

Finally, the presence of dextran increases the p*K*_a_ of a crucial GDH residue, while glucose does not (Table 1). Most likely, this residue is lysine 126, which must release a proton before the enzyme–substrate complex can close and undergo the essential H-transfer.^14^ Thus, the presence of dextran makes it more difficult for proton release from the crucial Lys to decrease GDH activity. The p*K*_a_ values of amino acid residues depend on their surroundings. The difference between the p*K*_a_ of the side chain fully solvated in water (the usual reference state) and the side chain present in the protein can be estimated by evaluating two major components: (i) dehydration and (ii) interaction with the protein environment, ions, or other components of the solution.^53–55^ The chemical nature of each type of crowder could influence the p*K*_a_, since, in principle, the crowder can directly interact with the Lys residues in the active site. However, this outcome was not observed in our MD simulations because dextran does not penetrate deeply enough into the active site. More likely, dextran indirectly influences the lysine p*K*_a_ by altering the protein conformation through excluded volume effects, which influences the electrostatic potential around Lys and leads to a change of its p*K*_a_.

This ability of a crowded environment to increase this p*K*_a_ from 7.8 to 8.4 is likely to have biological significance. While challenging to measure, the typical pH of resting mitochondria is estimated to be anywhere between 7.2 and 8.1,^56,57^ but the matrix pH increases significantly in an actively respiring mitochondria as protons are pumped out into the inner membrane space. The increased p*K*_a_ due to crowding requires the matrix to reach a higher pH before the GDH enzyme is fully active, allowing better control over GDH activity through a wider pH range. Both computational and experimental evidence supports the fact that cells may use crowding to exert physiological control and that the levels of matrix crowding fluctuate with mitochondrial stress.^58,59^ It is possible that cells indirectly regulate GDH through the level of macromolecular crowding, thereby further fine-tuning the GDH activity levels to respond to environmental conditions. Precedence exists in the literature for the claim that crowding may serve a regulatory role in cells to respond to environmental changes.^60^ In lower-level organisms, cells use crowding as a means to maintain physicochemical homeostasis through the use of biomolecular condensates.^61^ More recent evidence reveals that crowding can alter the allosteric regulation of the enzyme tyrosine phosphatase.^62^

While the effects from glucose may appear similar to those from dextran (Figure 2), the majority of the data in this study suggest that the mechanism by which dextran decreases GDH activity likely differs from that of its small-molecule counterpart. Unlike dextran, glucose is unable to increase the crucial p*K*_a_ (Table 1) and cannot promote the abortive GDH complex responsible for substrate inhibition (Figure 3). Furthermore, the effects from glucose are significantly less pH-dependent than those with dextran (Figure 4). The decreased GDH activity in the presence of glucose could likely result from an increased solution viscosity impeding product release from the GDH enzyme. Such an effect has been observed before for similar dehydrogenases, especially when a conformational change is required for product release.^63^ Alternatively, the effects may be due to the ability of glucose, as a small molecule, to penetrate the GDH active site and interact with the substrate (Figure S17). This explanation is consistent with the fact that glucose decreases GDH activity with glutamate but increases GDH activity with norvaline in lower pH solutions (Figure S5). The molecular dynamic simulations show that GDH with norvaline at pH 7 adopts a conformation different from that in the presence of glucose than in its absence (Figure S12), resulting in more flexibility of residues 200–300 (Figure S11). Previous studies have compared results with dextran to glucose, its small-molecule counterpart, to differentiate excluded volume effects from soft interactions;^28–31^ yet, our results show glucose can enter the GDH active site, while dextran cannot penetrate deeply into the interdomain region. These simulations show that small molecule “control” to differentiate soft interactions from excluded volume effects may not be appropriate with our current system. Nonetheless, the significant effect of glucose on the GDH kinetic parameters reveals the importance of considering cellular osmolyte concentrations for *in vitro* kinetic studies.

The cause for the significant decrease in enzymatic rate observed when GDH is premixed with glutamate remains unclear (Figure 5). It is tempting to suggest that this observation is related to substrate inhibition, since the preincubation more severely impedes GDH activity in the presence of dextran in a pH-dependent manner. Furthermore, the order that reagents are added to the norvaline assay has no effect on the resulting rates, and norvaline cannot form the abortive complexes. However, the fact that the preincubation uniformly decreases GDH activity in buffer regardless of pH (Figure 4 black) argues against substrate inhibition as its cause. Instead, it is possible that premixing GDH and glutamate enhances the coenzyme’s preference to bind to its regulatory site instead of the active site. Previous work shows that glutamate improves the coenzyme binding affinity at this regulatory site, thereby inhibiting GTP activity.^6^

## Conclusion

These experiments reveal how a crowded cellular environment may contribute to GDH regulation in a pH-dependent manner. Dextran appears to exert three main effects on GDH. First, dextran increases the p*K*_a_ of a crucial lysine residue, thereby impeding catalytic mouth closure necessary for the hydride transfer during catalysis. Second, crowding promotes a GDH-abortive complex, which increases glutamate substrate inhibition, but only at low pH values. Regardless of pH, dextran and other large crowders favor the closed GDH complex through excluded volume effects, thereby slowing product release.

Given the central metabolic role of GDH and the importance of its regulation, cells may be using the level of mitochondrial crowding as a means to adjust GDH activity in response to external factors. Despite previous controversy over the direction of the GDH-catalyzed reaction, experts now agree that *in vivo*, GDH mainly catalyzes the deamination of glutamate to supply alpha-ketoglutarate. This reaction provides citric acid cycle intermediates from an amino acid source rather than from carbohydrates or lipids. Under conditions when the energy charge (ATP/ADP ratio) of a cell is high, GDH activity should be inhibited so that glutamate can be shuttled to other uses, such as glutamine synthesis. As such, GDH has lower activity at low pH values below the crucial p*K*_a_ of the catalytic lysine. In this low pH environment, GDH is further impeded by cellular crowding, enhancing substrate inhibition. It is only as the cellular energy charge decreases to levels that initiate oxidative phosphorylation in the absence of carbohydrates and lipids that glutamate catabolism is necessary to supply citric acid cycle intermediates. This low energy charge activates the electron transport chain to actively pump protons, thereby increasing the pH of the matrix. The resulting pH increase improves GDH activity, as the crucial lysine residue becomes deprotonated and alleviates substrate inhibition. In addition, the crowded matrix environment allows for GDH to be sensitive to leucine activation, a signal of protein abundance, but only in high pH environments of presumably respiring mitochondria. In contrast, GDH needs to remain sensitive to GTP inhibition under all conditions as a signal of a high energy charge. In summary, the experimental and computational results presented here suggest that macromolecular crowding may function to modulate the GDH activity as an additional component of its already complex regulation.

## Methods

### Chemicals

D-(+)-glucose, sucrose, leucine, l-norvaline, l-glutamate, beef liver glutamate dehydrogenase (Roche, EC 1.4.1.3), Ficoll 70 (GE Healthcare), polyethylene glycol (PEG, 1 and 6 kDa; Calbiochem), polyvinylpyrrolidone (PVP, 40 kDa), bovine serum albumin (BSA), and dextran from *Leuconostoc mesenteroides* (∼9–11 and 150 kDa) and *Leuconostoc* spp. (450–650 kDa) were obtained from Millipore Sigma. Polyethylene glycol (300, 8000, and 20 000 Da from Alfa Aesar) was purchased from Fisher Scientific. Dried egg whites were purchased from Judee’s. Trehalose from Swanson was used for all experiments because trehalose from Sigma-Aldrich contained impurities that interfered with the kinetic assay (see section Controls). Solutions were prepared with 100 mM phosphate buffer (pH = 7.0) or 100 mM pyrophosphate buffer (varying pH) containing 10 μM EDTA. The crowding agent solutions were corrected to the appropriate pH before use.

### Kinetics Assays

Glutamate dehydrogenase (GDH) activity was determined by monitoring the increase in absorbance at 340 nm to detect the appearance of the NADH product every 8 s for 10 min with a Molecular Devices SpectraMax 190 instrument or every 22 s for 10 min with a Tecan Infinte M200 Pro spectrophotometer (Figure S18). Reactions were performed at 25 °C with shaking in a 96-well plate with a total volume of 200 μL. To minimize degradation, stock reagents were made fresh and kept on ice. When the glutamate or norvaline concentration was varied, each well contained 1 mM NAD^+^. When varying NAD^+^, each well contained 9 mM glutamate. The reaction was initiated by adding 50–150 nM GDH (depending on the solution conditions). Varying the enzyme concentration in this range did not change the effects of glucose or dextran on the kinetic parameters (Figure S5). Higher concentrations of enzyme (>150 nM) resulted in nonlinearity (Figure S18B,C) due to both a pre-steady-state burst and consumption of the substrate over time. All crowding assays were performed concurrently with an identical GDH assay lacking the crowder to minimize the variability.

### Michaelis–Menten Data Analysis

Initial enzymatic rates, *v*_0_, were obtained by taking the best-fit average slope from absorbance vs time plots using SoftMax Pro 6.3 or Magellan software (Figure S18). Three initial rates were obtained per reaction condition and averaged. These averaged initial rates were plotted against substrate concentrations, [*S*], to construct a Michaelis–Menten curve of 9 data points (Figure S19). Then, SigmaPlot was used to obtain the Michaelis constant (K_m_) and the maximum rate (*V*_max_) from best fits to the equation:

1 $$v_{0}=\frac{V_{max}\left[S\right]}{K_{m}+\left[S\right]}$$

K_m_ and *V*_max_ values obtained from crowded conditions were divided by the values obtained on the same 96-well plate in buffer only, yielding relative kinetic values. This process was repeated in triplicate (or as indicated in the figure) with independent reagents, yielding average relative kinetic values.

To investigate substrate inhibition, higher concentrations of glutamate (0–25 mM) were used, and the resulting curve was fit to the equation:

2 $$v_{0}=\frac{V_{max}}{1+\frac{K_{m}}{\left[S\right]}+\frac{\left[S\right]}{K_{i}}}$$

where K_i_ is the inhibition constant.

### Controls

GDH assays, in the absence of glutamate, yielded flat Abs_340_ vs time slopes that were unaffected by the addition of 10 μM leucine, confirming that leucine is a poor substrate for GDH.^49^

Furthermore, synthetic crowding agents like dextran can contain impurities that interfere with enzyme assays.^63^ All crowding agents used in this study passed a “negative control” where the GDH kinetic assay was performed in the presence of the crowder with one reagent omitted (enzyme, NAD^+^, or glutamate) and no enzyme activity (<1% compared to the slopes of the complete assay) was detected. BSA from Thermo Fisher Scientific, trehalose from Sigma-Aldrich, and lysozyme each failed this control and thus were not used in further experiments. Polyethylene glycol (PEG) 100,000 and polyvinylpyrrolidone (PVP, 10 kDa) inhibited all GDH activity (Figure S20).

### Varying pH

When varying the pH of the solution, phosphate buffer was replaced by pyrophosphate, given its relevant p*K*_a3_ = 6.6 and p*K*_a4_ = 9.4 values to maintain a wider range of buffering capacity.^64^ It is important to note that GDH activity is lower in pyrophosphate than in phosphate buffer (Figure S21), but the same crowding effects were observed regardless of the buffer (Table S3). Assays were run using 0.1 M pyrophosphate containing 10 μM EDTA, 1.0 mM NAD^+^, 9 mM glutamate or 150 mM norvaline, and 150 nM GDH. To obtain the p*K*_a_ of the relevant residue in GDH, initial rates (*v*_0_) vs pH values were plotted, and the resulting curve was fit using Sigma Plot to the equation:

3 $$v_{0}=\frac{v_{0,max}}{1+10^{(pK_{a}-pH)}}$$

where *v*_0,max_ is the maximum initial velocity at the optimal pH value.

### Premixing (Altering the Order of Adding Reagents to Kinetic Assay)

150 nM GDH was mixed with 1 mM NAD^+^ in 100 mM pyrophosphate buffer with 10 mM EDTA and allowed to incubate for 10 min before 9 mM glutamate or 150 mM norvaline was added to initiate the reaction. Alternatively, 120 nM GDH was mixed with 9 mM glutamate or 150 mM norvaline and allowed to incubate for 10 min before 1 mM NAD^+^ was added to initiate the reaction.

### Temperature

Using 9 mM glutamate or 150 mM norvaline, 120 nM GDH, and 1 mM NAD^+^ in 100 mM pyrophosphate buffer with 10 μM EDTA, kinetic assays were completed as a function of temperature between 20 and 40 °C. The slope of the resulting absorbance vs time plot was divided by the enzyme concentration to yield the turnover number, *k*_cat_, and the resulting Eyring plots (Figure S8) were fit to the following equation to determine the enthalpy (ΔH^⧧^) and entropy (ΔS^⧧^) of activation:

4 $$\ln\left(\frac{k}{T}\right)=\frac{-\Delta H^{⧧}}{R}\frac{1}{T}+\ln\left(\frac{k_{B}}{h}\right)+\frac{\Delta S^{⧧}}{R}$$

### Molecular Simulations

The structure of bovine glutamate dehydrogenase trimer was obtained from the RCSB PDB database (PDB ID: 6DHQ).^65^ This crystal structure contains the hexamer (dimer of trimers) with glutamate, GTP, and the reduced NADPH. Due to the computational cost, we used just the trimer (Figure 1). Depending on the composition of the simulated model system (see Table S4), the substrates were kept, removed, or altered by norvaline or NAD^+^ in order to better mimic the experimental setup. Missing hydrogen atoms were added employing the GROMACS pdb2gmx tool.^66^ The protonation states of titratable amino acid residues at pH = 7 were determined using the ProToss web server.^67,68^ The p*K*_a_ values were also predicted by the PROPKA 3.4.0 software,^53,54^ which indicated values below 9 for the three Lys residues in the active site (Lys 90, 114, and 126). Thus, to simulate pH = 7, these three Lys were protonated, and at pH = 9, neutral (Figure S22).

The protein was described by the Amber ff99SB*-ILDN force field,^69–71^ Na^+^ counterions using the default parameters included in the ff99SB*-ILDN force field, and water by the TIP3P water model.^72^ The force field parameters for the reactants were adopted from http://amber.manchester.ac.uk/. Glucose was described by employing a modified version^73^ of the GLYCAM06 force field,^74^ featuring adjusted intermolecular Lennard-Jones interactions to prevent excessively attractive sugar–sugar and amino acid–sugar interactions. We recently utilized such successful glucose parametrization to construct and validate the force field for dextran 10,^75^ which was employed in the current study.

The glutamate dehydrogenase trimer with the substrates (if applicable) was placed in a cubic box of roughly 15 nm. The glucose molecules were inserted around the protein using the PACKMOL software^76^ at the 100 g/L concentration. The same concentration of the dextran10 polymer chains was generated employing the polyply gen_coords tool.^77^ The resulting systems were then relaxed, hydrated, and equilibrated in the same way as in our dextran10 simulation study.^75^ The model system is depicted in Figure S23.

The all-atom molecular dynamics trajectories were collected using the GROMACS software (version 2020.4).^66^ The Newton’s equations of motion were integrated with a time step of 2 fs using the leapfrog algorithm.^78^ The lengths of all hydrogen-containing solute bonds were constrained by the LINCS algorithm,^79^ and the internal geometry of water molecules was kept rigid by the SETTLE algorithm.^80^ Short-range electrostatic and van der Waals interactions were treated with a 1.2 nm cutoff, while long-range electrostatic interactions were evaluated using the particle mesh Ewald method.^81^ The velocity rescaling thermostat with a stochastic term^82^ with a time constant of 1 ps maintained the temperature of the system at 300 K. The Parrinello–Rahman barostat^83^ with a time constant of 1 ps was used to keep the pressure at 1.01 bar. The length of the simulations was 200 ns for the dilute and glucose-containing systems and 400 ns for the dextran-containing systems.

The resulting trajectories were visualized in the VMD software.^84^ The root-mean-square deviation of the Cα atoms with respect to the reference crystal structure and the root-mean-square fluctuation of the Cα atoms were calculated using the GROMACS toolbox.^66^ The first 100 ns of the trajectories of the dextran-containing systems were discarded from the analysis of the time-averaged properties (RMSF) because of the anticipated need for longer equilibration. The angle between the NAD- and GLU-binding domains was determined as follows. We first determined the principal axis of each domain (residues 200–370 and 1–199, respectively). This axis represents the longest dimension of a best-fit ellipsoid that describes the domain’s shape. It is derived from the mass-weighted distribution of the atomic positions.

## Supplementary Material



## Notes

Relative *V*_max_ and K_m_ values in the presence of PEG, BSA, Ficoll, sucrose, glucose, various sizes of dextran, and binary crowding mixtures; GDH denaturation curves in BSA, glucose, or dextran; pH dependence of GDH activity at varying enzyme concentrations, different orders of addition of substrates, or with norvaline; Eyring GDH plots; example raw data, including absorbance vs time plots for the GDH assays, representative Michaelis–Menten curves for varying glutamate in the presence of PEG, leucine, GTP, BSA, glucose, or dextran; table of *V*_max_ and K_m_ at varying temperatures, in the presence of GTP, leucine, glucose, and dextran, or in a different buffer (phosphate vs pyrophosphate); GDH activity at increasing concentrations of bithionol, zinc, or PVP; molecular dynamics simulations of dextran or glucose with GDH; RMSF and RMSD for C_α_ of GDH; pH-dependent arrangement of substrates in the GDH active site; snapshots of substrates leaving the active site (PDF)

## Abbreviations

BSA: bovine serum albumin (protein crowder)
dex: dextran (the number afterward represents the molecular weight of the polymer in kDakDa
GDH: glutamate dehydrogenase
GTP: guanosine triphosphate
kDa: kilodaltons
K_m_: Michaelis constant
NADH: nicotinamide adenine dinucleotide
PEG: polyethylene glycol
PVP: polyvinylpyrrolidone
*V*_max_: maximal rate under Michaelis–Menten kinetics
