# Supplementary material for: Can Macromolecular Crowding Help Regulate Glutamate Dehydrogenase Activity?
Source: ACS Omega. 2025 Oct 31;10(44):53060–73. doi: 10.1021/acsomega.5c07618 (PMC12612887; doi:10.1021/acsomega.5c07618)
Supplement: Supplementary file 1 [file ao5c07618_si_001.pdf]

Supplemental Information for

Can Macromolecular Crowding Help Regulate Glutamate Dehydrogenase Activity?

*Genesis Rosario,[1] Andrea Desrochers,[1] Alec Robitaille, [1] Emily Rundlett, [1] Daniel Myšák, [2] Zuzana Sochorová Vokáčová,[2] Štěpán Timr, [2] Eva Pluhařová,[2] and Kristin M. Slade[1]**

1. Department of Chemistry, Hobart and William Smith Colleges, Geneva, NY 14456
   300 Pulteney St
   Geneva NY 14456
2. J. Heyrovský Institute of Physical Chemistry of the Czech Academy of Sciences,
   v. v. i., Dolejškova 2155/3, 182 23 Prague 8, Czech Republic

* Corresponding author: slade@hws.edu

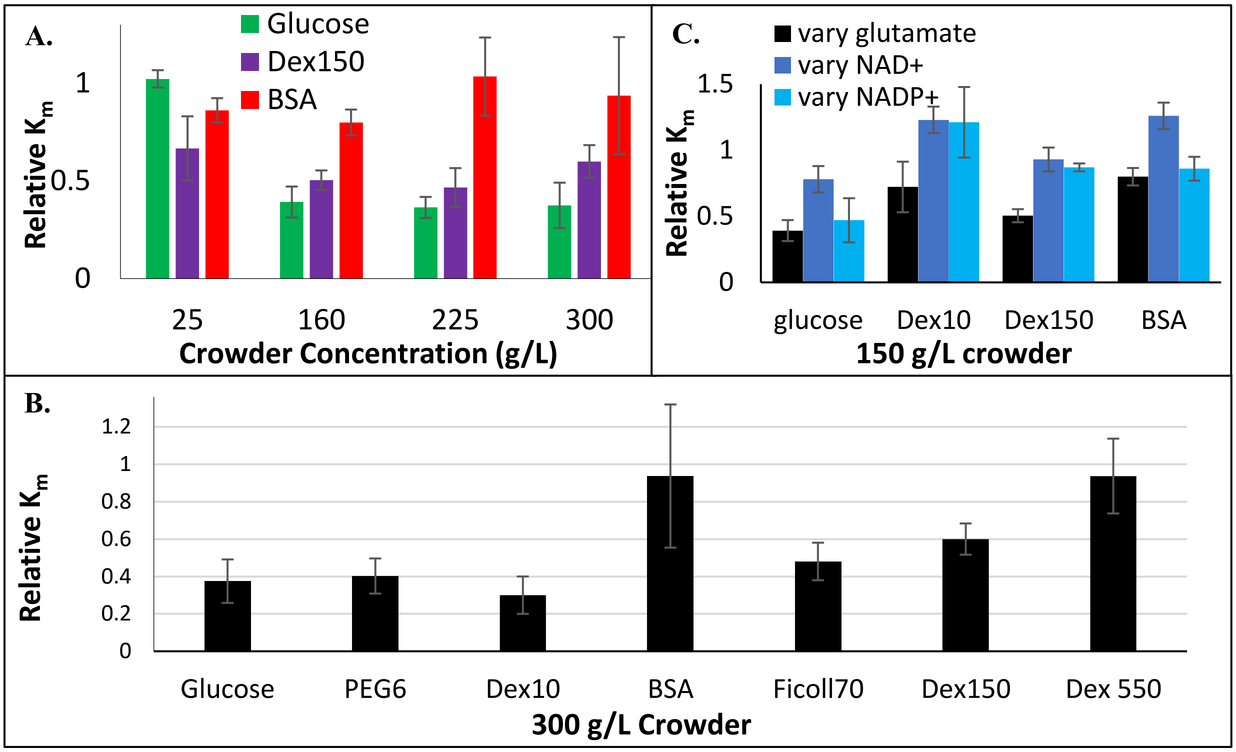

Figure S1. Effects of Crowding on the GDH Michaelis-Menten Contant. 60 nM GDH assays in 100 mM phosphate buffer (pH =7.0) with A-B) 1 mM NAD^+ and varying glutamate concentrations, or C) 20 mM glutamate at varied NAD^+ or $NADP^+$ concentrations were performed in the presence of A) varying concentrations, B) 300g/L, or C) 150 g/L crowder. K_m values from the resulting Michaelis−Menten curves were normalized to values acquired in buffer only to yield "relative K_m values. Error bars represent standard deviations (n = 3).

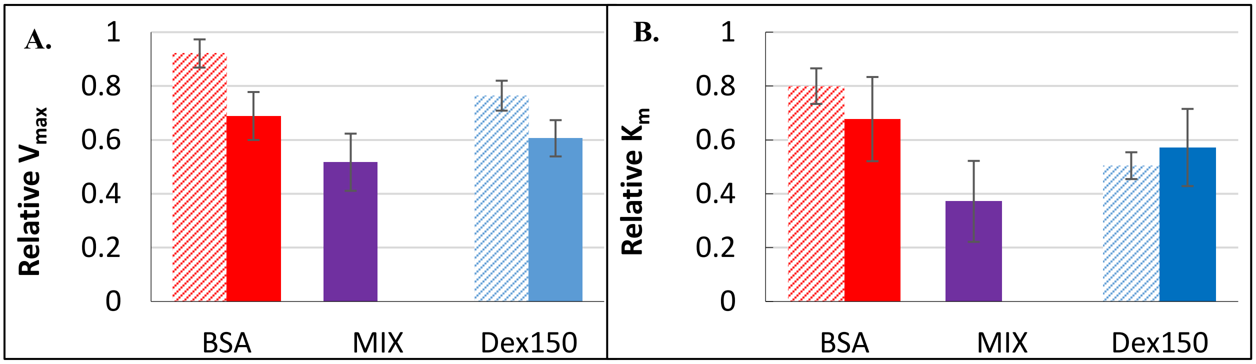

Figure S2. Effects of Crowding Mixtures GDH kinetics. Assays in 100 mM phosphate buffer (pH = 7.0) at 1 mM NAD^+, 60 nM GDH and varying glutamate concentrations were performed in the presence of 300g/L (solid bars) or 150g/L (striped bars) dextran 150 (blue) or BSA(red). "MIX" (purple) is a mixture of two crowders each at 150 g/L. A) V_{max} and B) K_m values from the resulting Michaelis−Menten curves in Crowder were normalized to values acquired in buffer only to yield "relative V_{max} and K_m" values. Error bars represent standard deviations (n = 3).

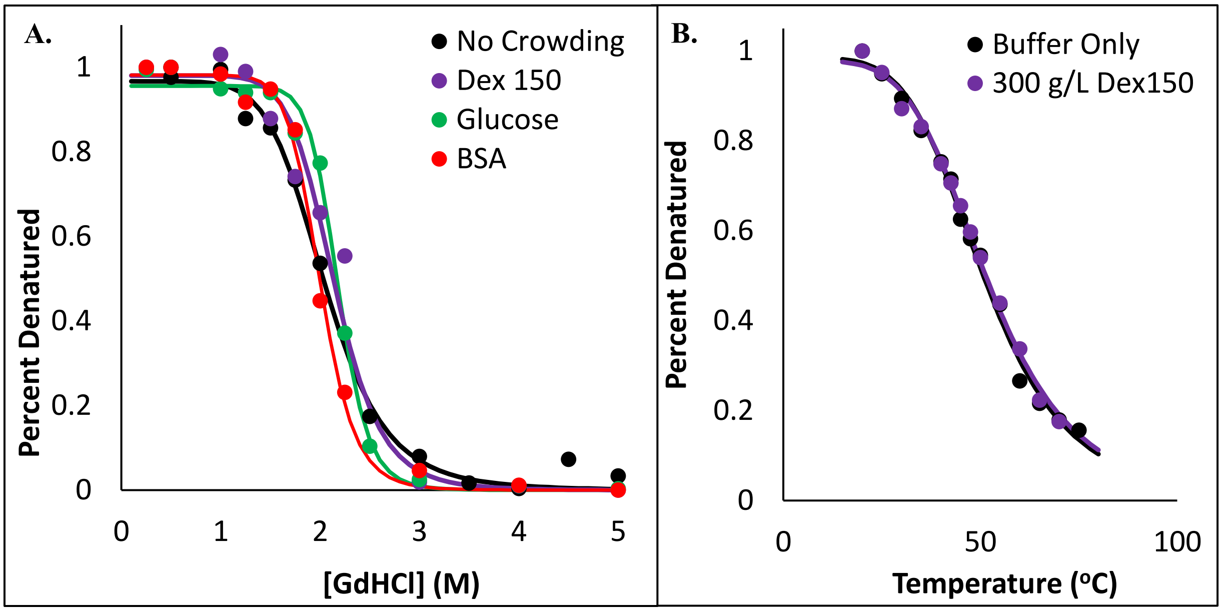

Figure S3. GDH denaturation. GDH was incubated for A)1 hr with various concentrations of guanidinium hydrochloride or B) 20 min at elevated temperatures in the presence of 300 g/L glucose (green), dextran 150 kDa (purple), bovine serum albumin (BSA, red) or buffer only (black circles).

Methods for Denaturation Studies: Solutions containing 370 nM g/L GDH and 300 g/L dextran 150 kDa, BSA, or glucose were incubated with 0 to 5.0 M guanidine hydrochloride (GdHCl) for 1 h at 25°C, before analysis with a Cary Eclipse fluorimeter (Agilent) with excitation and emission slits set to 10 nm. To monitor the resulting tryptophan fluorescence, the excitation wavelength was 295 used to collect emission spectra from 300-400 nm. The fraction of GDH denaturation, f_D was quantified as previously described[1] using:

$$f_D = \frac{I - I_N}{I_U - I_N} \quad (3)$$

where I_N is the fluorescence intensity of the native state without denaturant, I_u the fluorescence intensity of the fully unfolded state, and I is the measured fluorescence intensity at each GdHCl concentration.

Temperature unfolding of GDH was monitored by the absorbance of aromatic amino acids at 280 nm on a PerkinElmer spectrophotometer. Experiments were completed in the presence or absence of 300 g/L dextran 150 kDa and 600 nM GDH in 0.1 M phosphate buffer at a pH of 7. Temperature was increased from 20°C to 80°C, at 5 degree increments and samples were allowed to equilibrate for 20 minutes at each temperature. Data was analyzed by assuming initial absorbance levels at 20°C corresponded to native state (fully folded) and dividing subsequent points by the initial fluorescence measurement to give a percent denatured value.

Reference:

[1] Ahmad, F., Yadav, S., and Taneja, S. (1992) Determining stability of proteins from guanidinium chloride transition curves, *The Biochemical journal 287 (Pt 2)*, 481-485.

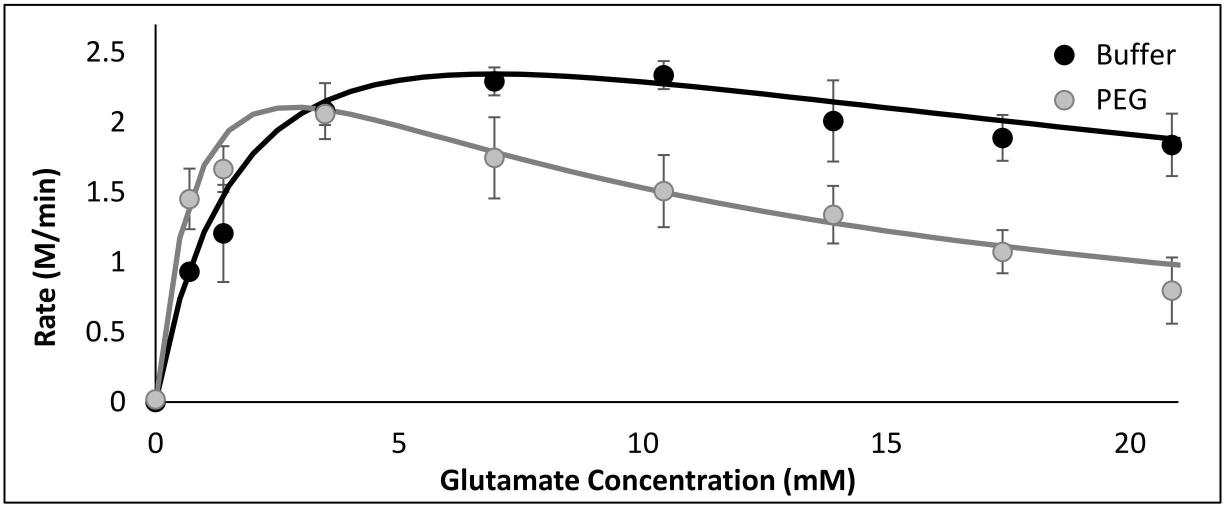

Figure S4. PEG enhances GDH substrate inhibition Assays containing 1 mM NAD^+, 150 nM GDH and varying glutamate concentrations were run in 100 mM phosphate buffer (pH = 7.0) in the absence (black) or presence of 200 g/L PEG 1000 (grey). The resulting data was fit to The Michaelis Menten equation modified for substrate inhibition (Eq 2.) Error bars represent standard deviations (n = 3).

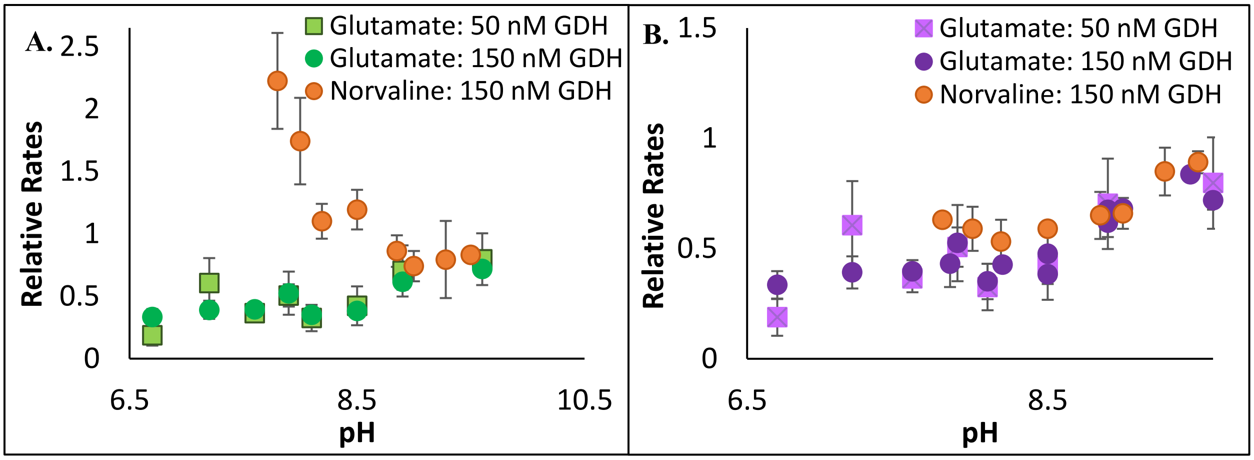

Figure S5. pH effects from glucose and dextran with glucose or norvaline. independent of enzyme At 9 mM glutamate and 1 mM NAD^+, reaction rates were measured for 50 nM (squares) or 150 nM (circles) GDH in the presence of 300 g/L A) glucose or B) Dextran 150 kDa in 100 mM pyrophosphate buffer. Alternatively, 180 mM norvaline was used in place of glutamate (orange circles) at 150 nM GDH. For each substrate, initial rates measured in the presence of dextran or glucose were divided by rates acquired in buffer only to yield relative rates. Error bars represent standard deviations (n = 3).

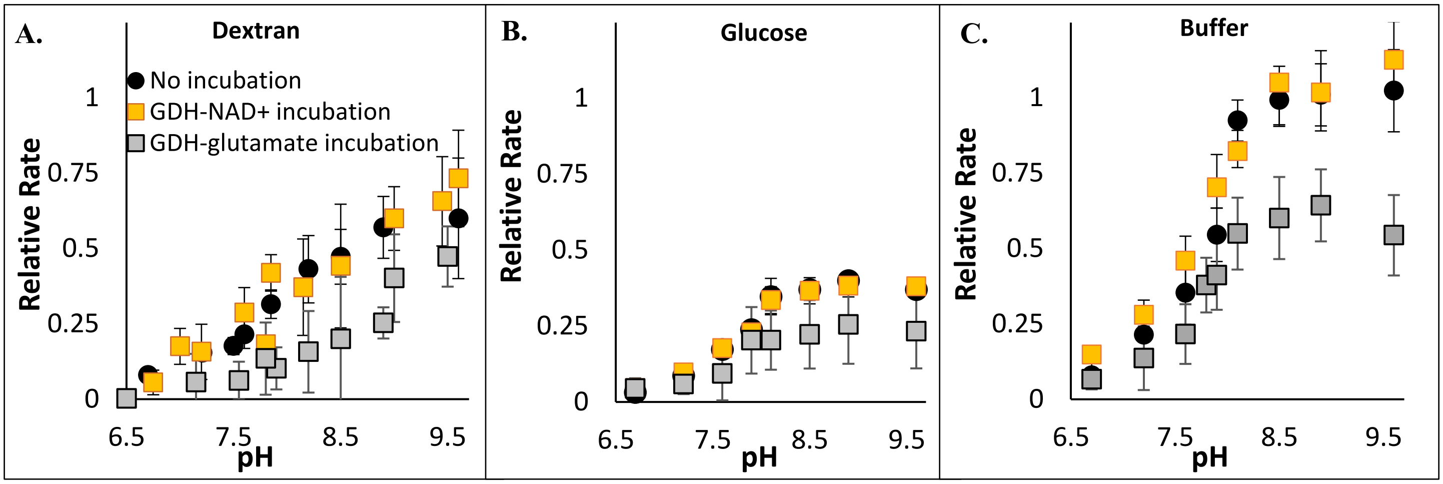

Figure S6. Order of addition matters for pH-dependence of crowding effects. GDH kinetic assays were run in varying pH buffers in the presence of A) 300 g/L dextran 150 kDa, B) 300 g/L glucose (green) or C) no crowder (buffer only). Each assay contained 150 nM GDH, 9 mM glutamate and 1 mM NAD^+, but the order of addition differed. GDH was added last (150 nM) to initiate the reaction (black circles), or GDH was pre-incubated with glutamate for 10 min before adding NAD^+ to initiate the reaction (grey squares), or GDH was pre-incubated with NAD^+ for 10 min before adding to glutamate initiate the reaction (orange squares). Error bars represent standard deviations (n = 3).

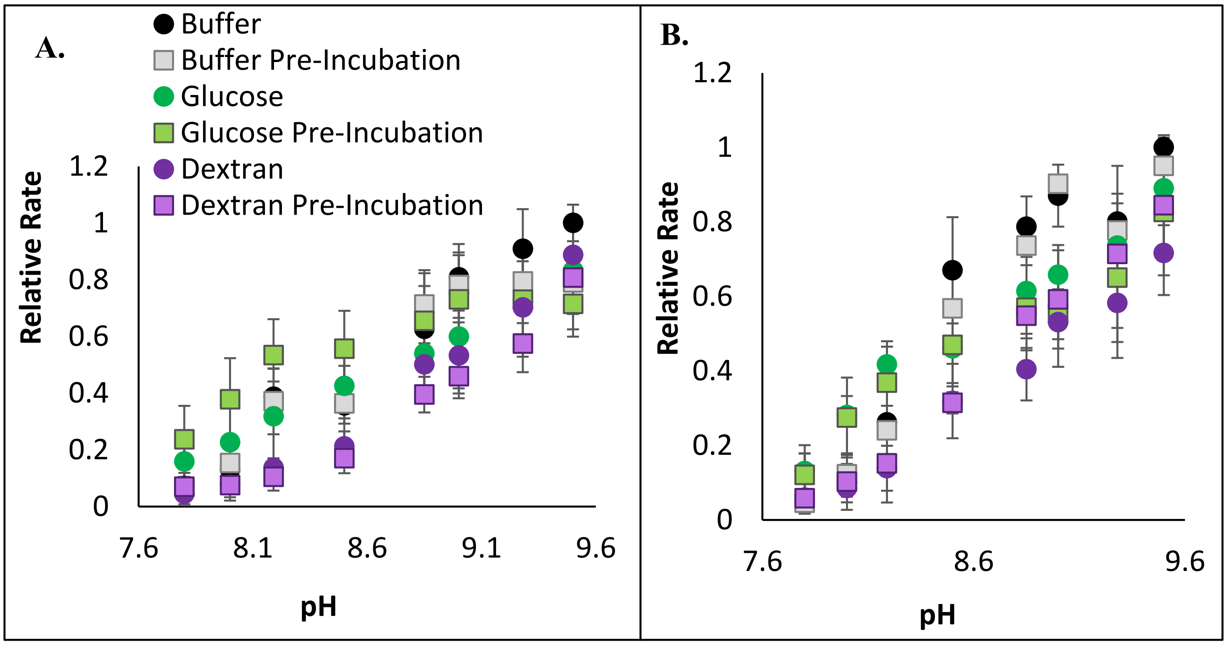

Figure S7. Pre-mixing of GDH with norvaline. GDH kinetic assays were run in varying pH buffers in the presence of 300 g/L glucose (green) or dextran 150 kDa (purple). GDH was added last to initiate the reaction (circles) or GDH was pre-incubated with A) norvaline for 10 min before adding 1 mM NAD^+ to initiate the reaction (squares). B) GDH was pre-incubated with 1 mM NAD^+ for 10 min before adding to glutamate initiate the reaction (squares). Error bars represent standard deviations (n = 3).

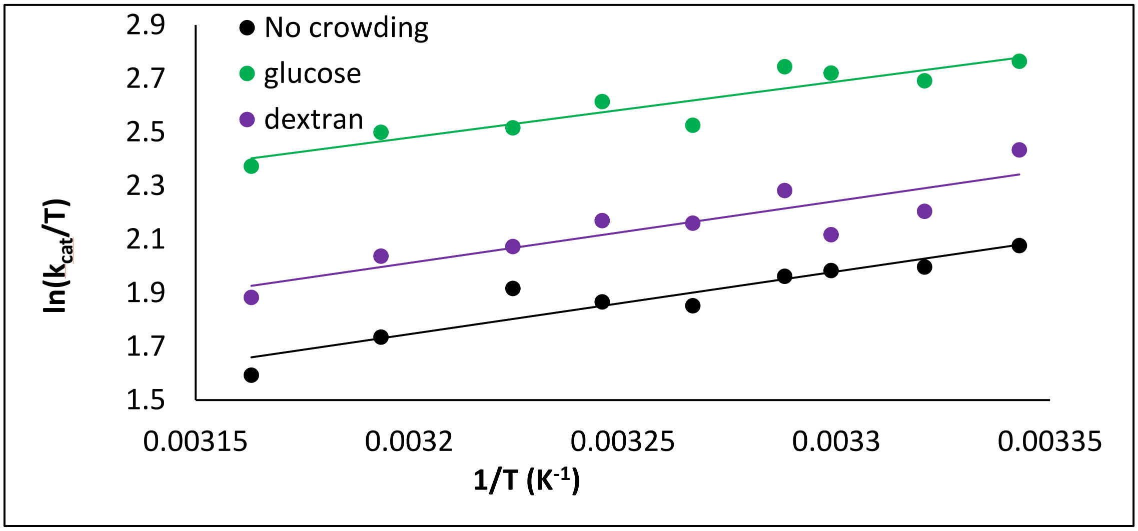

Figure S8. Eyring Plots for GDH. Assays were run in the absence (black) or presence of 300 g/L glucose (green) or dextran 150 kDa (purple) with 9 mM glutamate, 1 mM NAD^+ in 100 mM phosphate buffer (pH = 7.0) and 10 µM EDTA. at a function of temperature. Initial rates were divided by enzyme concentration to determine k_{cat}.

Table S1. Temperature influence on crowding effects of GDH kinetics at pH =7

Relative V_{max}	25°C	37°C
Glucose	0.54 ± 0.09	0.47 ± 0.1
Dextran	0.48 ± 0.09	0.53 ± 0.1

Relative K_m	25°C	37°C
Glucose	0.3 ± 0.1	0.3 ± 0.1
Dextran	0.6 ± 0.1	0.5 ± 0.2

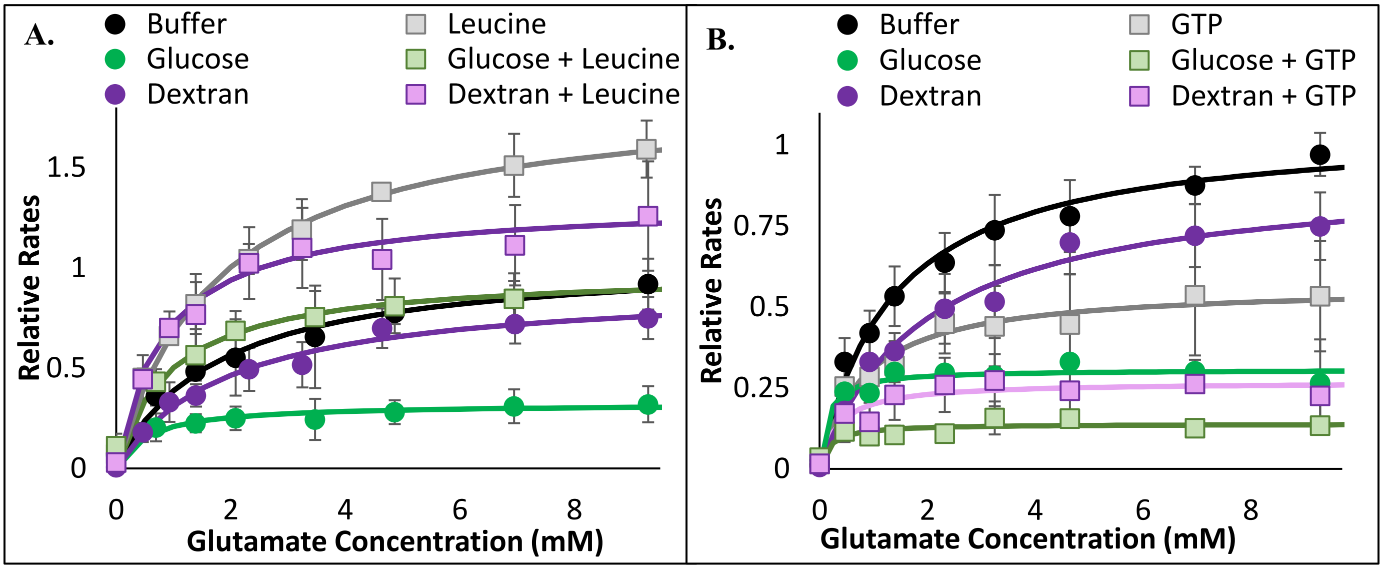

Figure S9. GDH regulation Kinetic assays A) with 10 µM leucine or with 10 µM GTP were run with 1 mM NAD^+, 60 nM GDH, 9 mM and glutamate with 300 g/L for glucose or dextran 150 in 0.1 M pyrophosphate buffer (pH = 9) with 10 µM EDTA. Error bars represent standard deviations (n = 3).

Table S2. Michaelis Menten Constants (K_m) of GDH kinetics at pH =9*

	K_m (mM)	K_m (mM) in Leucine	K_m (mM) in GTP
**Buffer**	1.8 ± 0.1	1.7 ± 0.2	0.8 ± 0.1
**Glucose**	0.53 ± 0.05	1.0 ± 0.1	0.2 ± 0.1
**Dextran**	1.1 ± 0.2	0.8 ± 0.1	0.3 ± 0.1

*values based on the curves in Fig S9

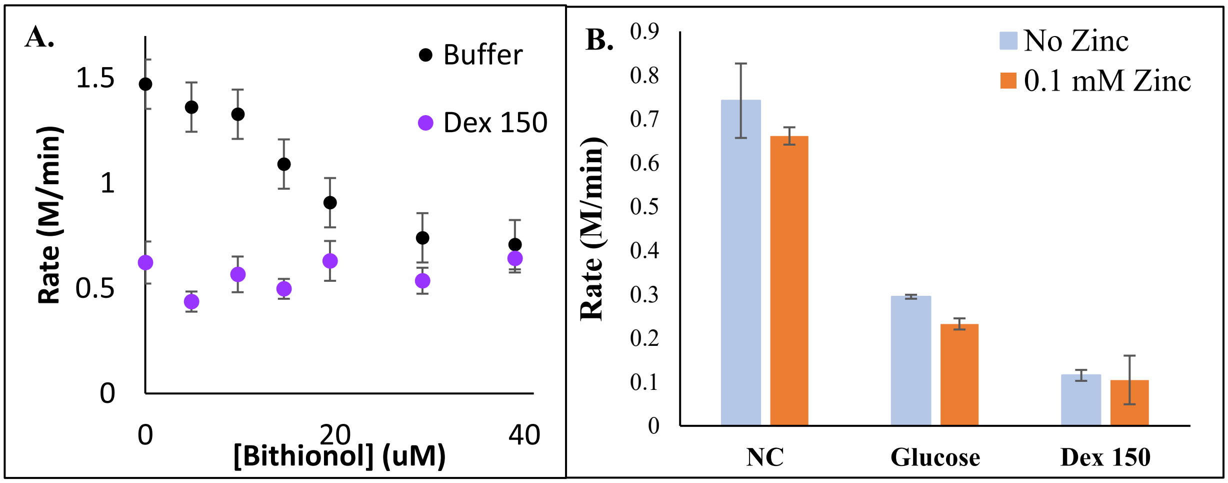

Figure S10. Effects of crowding on GDH inhibition Kinetic assays A) with Bithionol or B) with zinc chloride were run with 1 mM NAD^+, 60 nM GDH, 9 mM and glutamate with 300 g/L for glucose or dextran 150 in 0.1 M phosphate buffer (pH = 7) with 10 µM EDTA. Error bars represent standard deviations (n = 3).

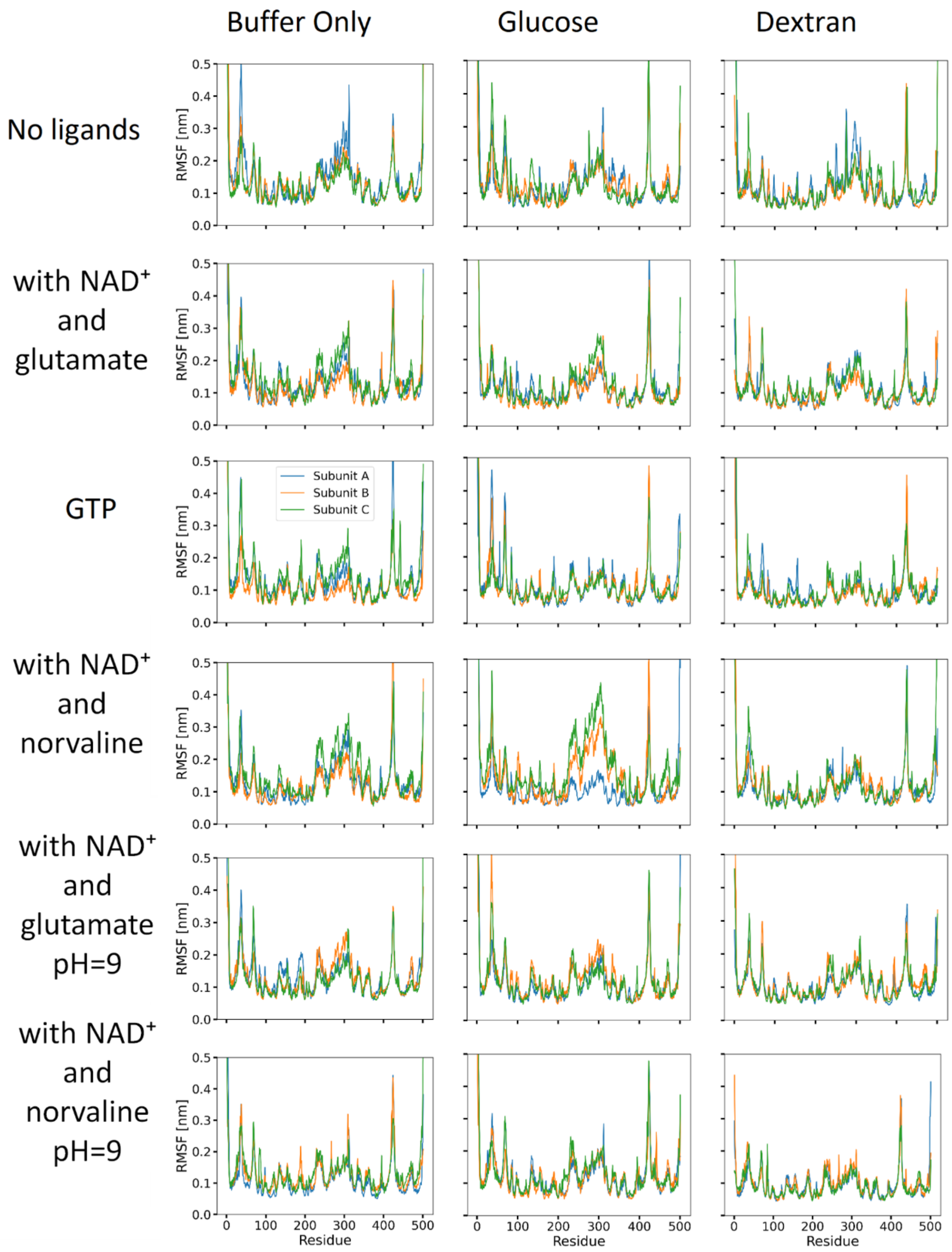

Figure S11. **Root-mean-square fluctuations of the protein Cα- atoms.** These values were calculated for glutamate dehydrogenase trimer complex (in rows: 1. protein without substrates, pH = 7, 2. protein with NAD^+, GLU, pH = 7, 3. protein with NADPH, GTP, GLU pH = 7, 4. protein with NAD^+, norvaline, pH = 7, 5. protein with NAD^+, GLU, pH = 9, 6. protein with NAD^+, norvaline, pH = 9, in columns: 1. water, 2. 100g/L glucose in water, 3. 100g/L dextran in water). Different colors correspond to different subunits (A blue, B orange, C green).

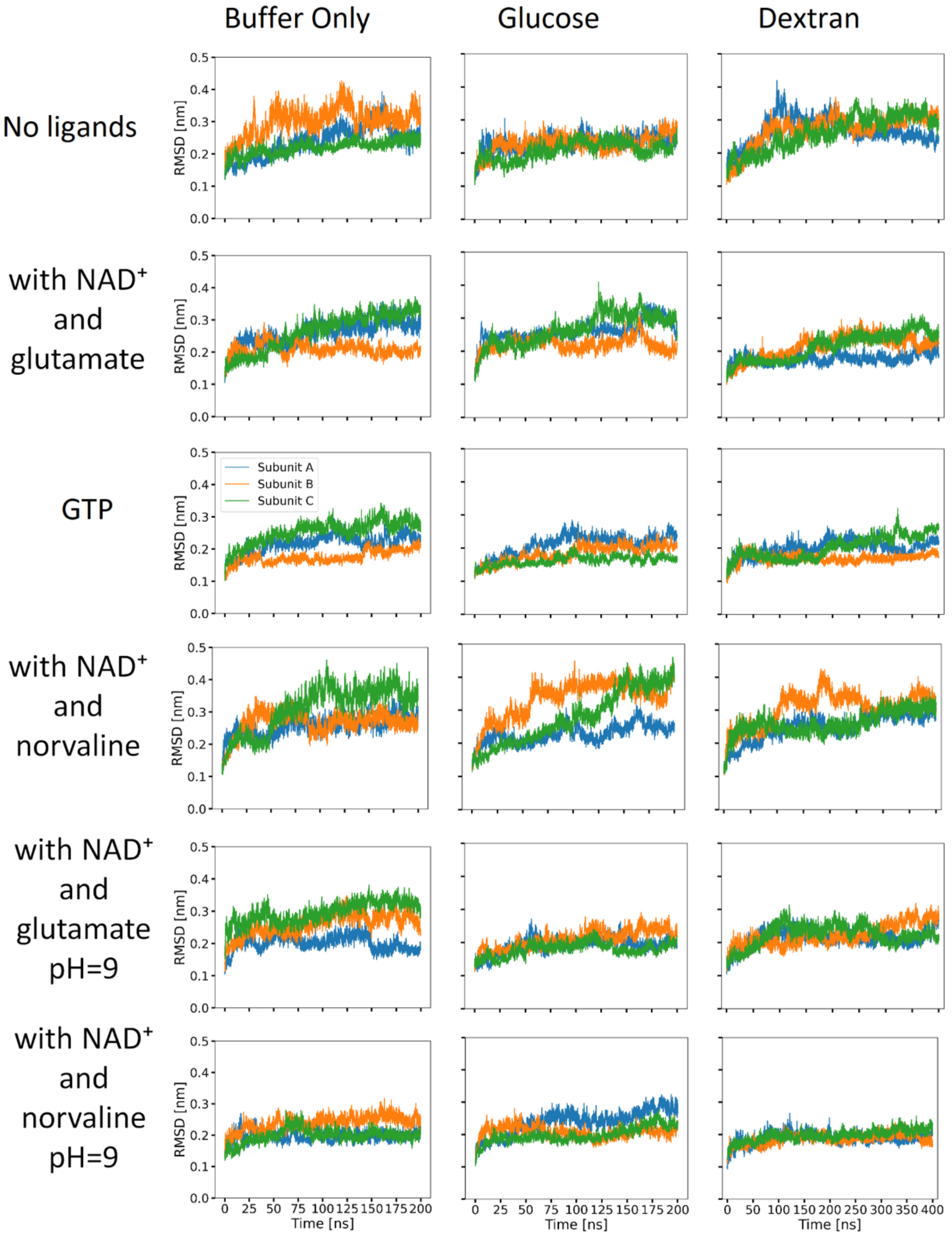

Figure S12. Time evolution of the root-mean-square deviation of the protein Cα atoms with respect to the crystal structure calculated for glutamate dehydrogenase trimer complex (in rows: 1. protein without substrates, pH = 7, 2. protein with NAD^+, GLU, pH = 7, 3. protein with NADPH, GTP, GLU pH = 7, 4. protein with NAD^+, norvaline, pH = 7, 5. protein with NAD^+, GLU, pH = 9, 6. protein with NAD^+, norvaline, pH = 9, in columns: 1. water, 2. 100g/L glucose in water, 3. 100g/L dextran in water). Different colors correspond to different subunits (A blue, B orange, C green).

A.

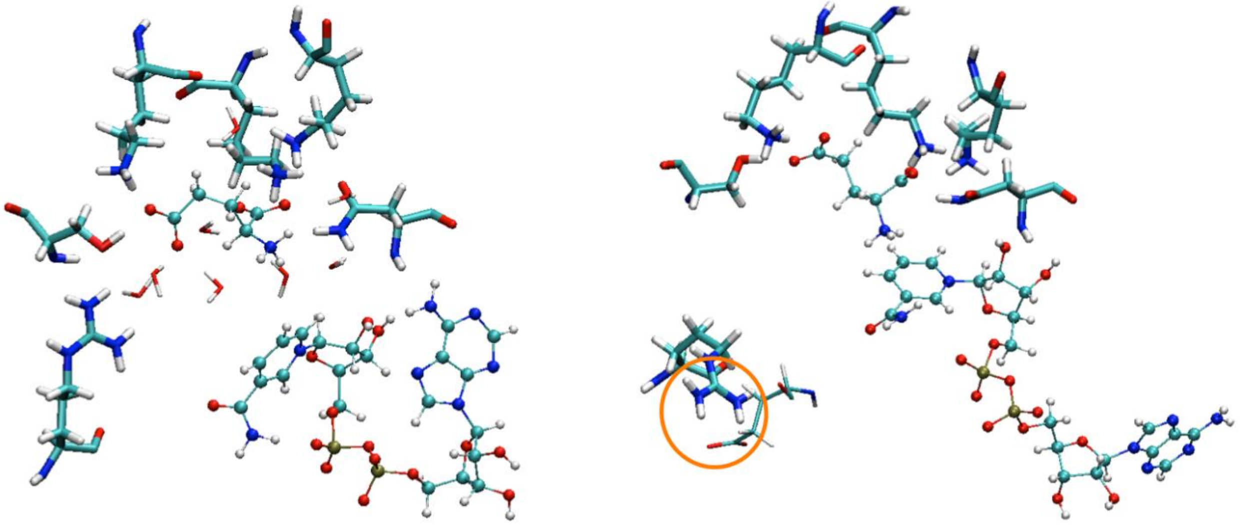

B.

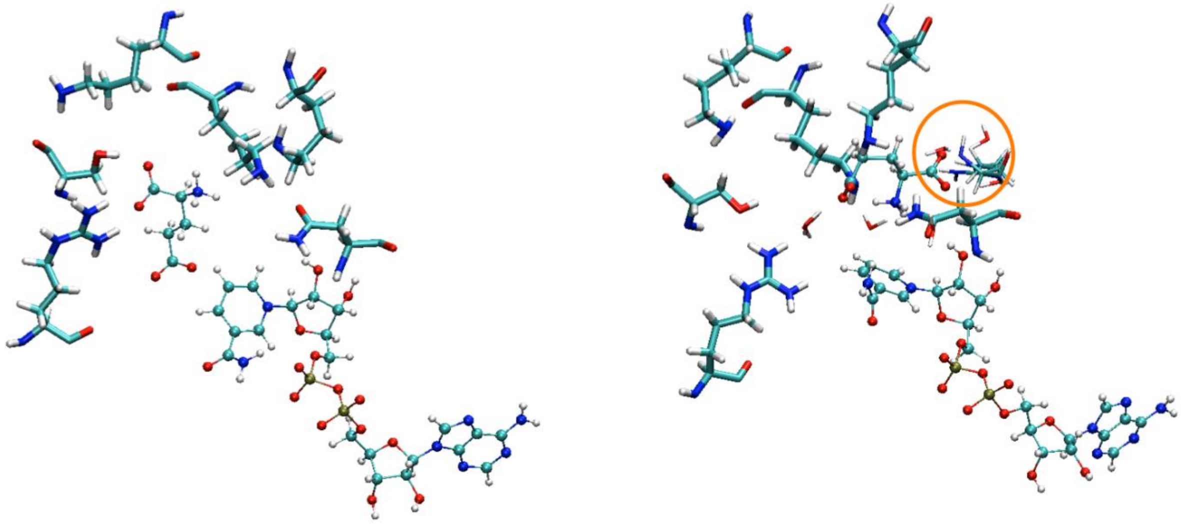

Figure S13.**pH-dependent arrangements of glutamate in the active site of GDH.** A) At pH 7, water molecules can penetrate between the substrate and NAD^+ and even Arg211 and glutamate (left). Side chain of Arg211 is flexible (right) and can form a salt bridge with Glu173 (orange circle). B) At pH 9, glutamate adopts alterternative conformation where the α-COO^- of glutamate can salt bridge with Arg211(left) or is attracted towards Arg94 (orange circle, right)

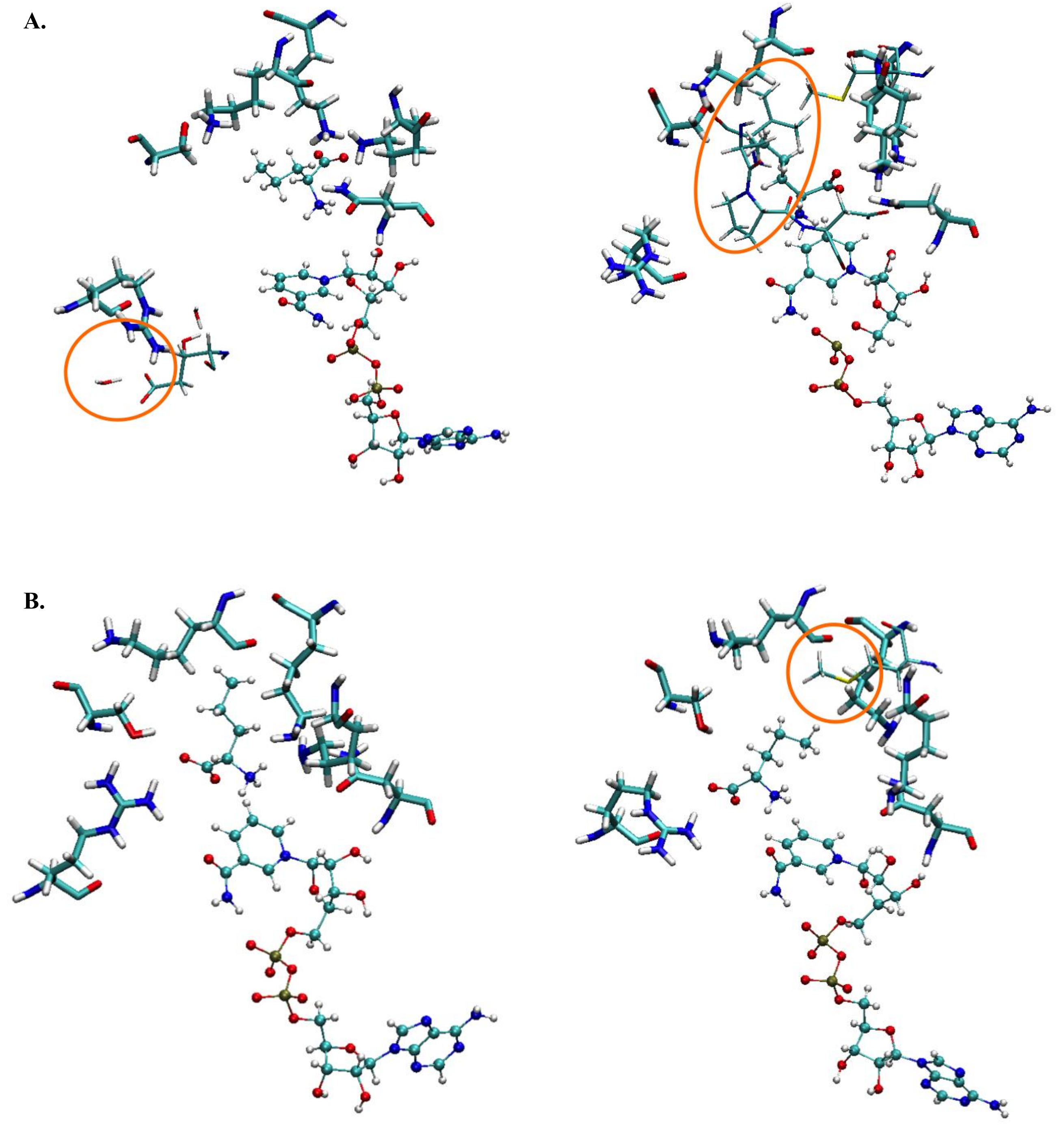

Figure S14. pH-dependent arrangements of norvaline in the active site of GDH. A) At pH = 7 with $-COO^{-}$ interacting with protonated $-NH_3^{+}$ Lysine residues can salt-bridge with Arg211-Glu173 (orange circle on the left). Other potential arrangement with nonpolar cavity Val378, Met111, Ala166, Pro167 (orange circle on the right). B) at pH = 9, where nonpolar part of norvaline is in the vicinity of nonpolar parts of lysine residues (left) or in contact with Met111 (orange circle right).

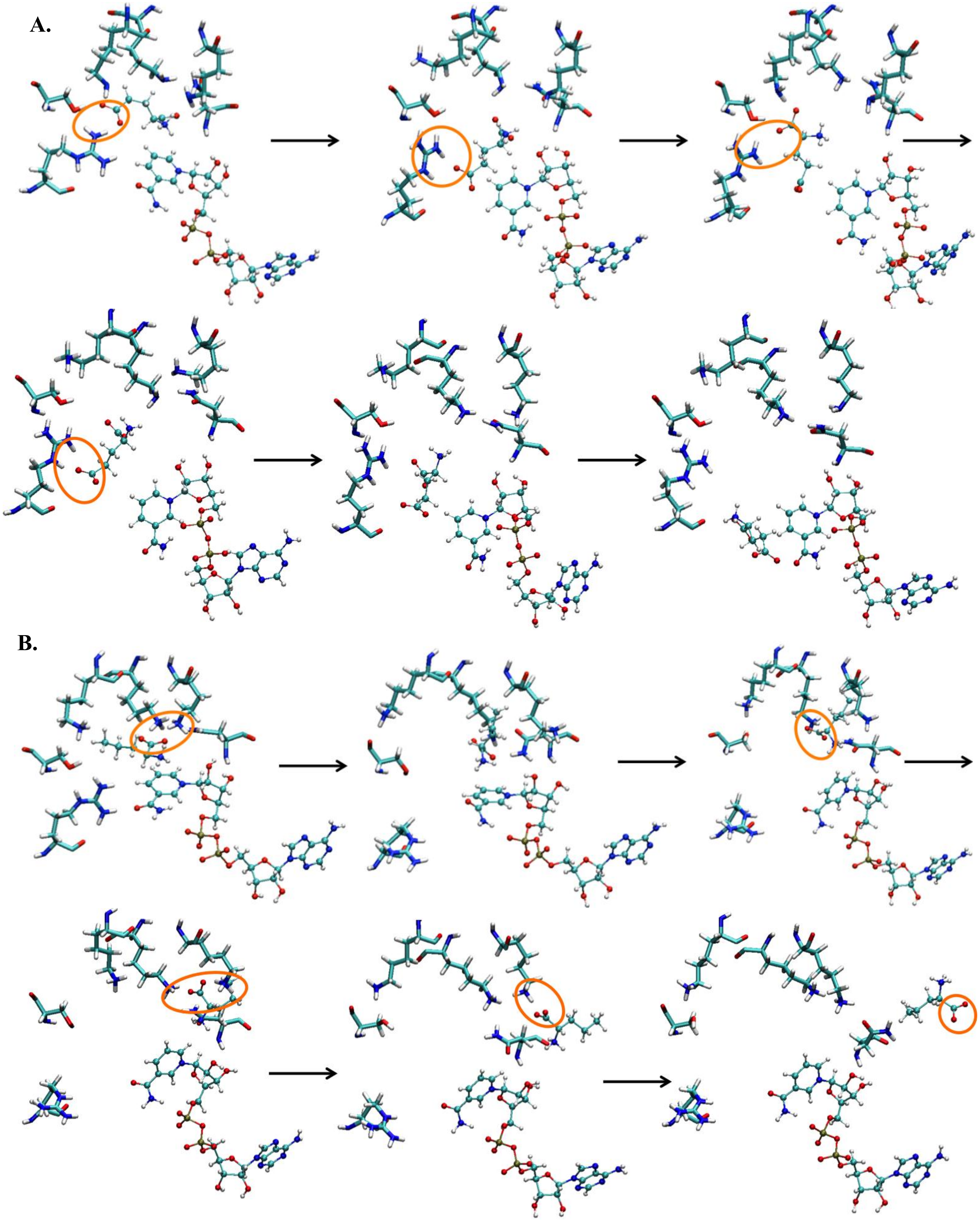

Figure S15. Spontaneous leaving pathway for substrates from the GDH active site. A) Monitoring glutamate leaving from the active site at pH = 9 over time. B) Monitoring norvaline leaving from the active site at pH = 7. Orange circles indicate the carboxylic group and potential salt-bridges formed between A) glutamate and Arg side chain or B) norvaline and Lys.

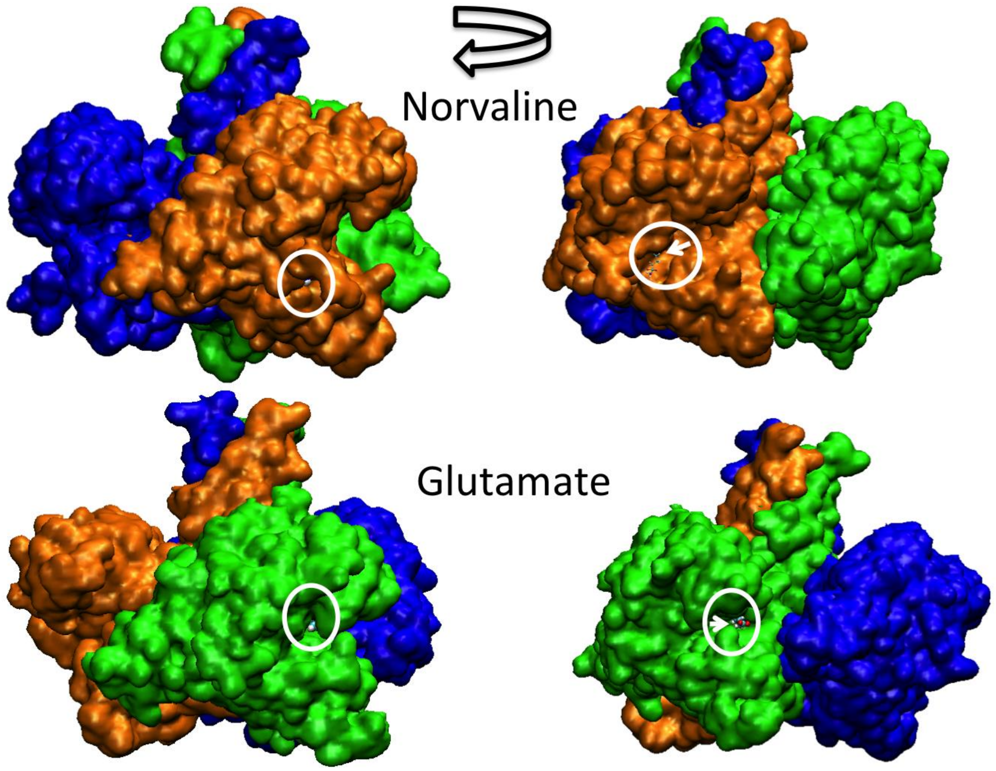

Figure S16. Spontaneous leaving pathway for GDH substrates. The place of the protein surface where substrate leaves (while circles). Norvaline leaves in the direction to the bulk (white arrow), while glutamate leaves towards other subunit (white arrow).

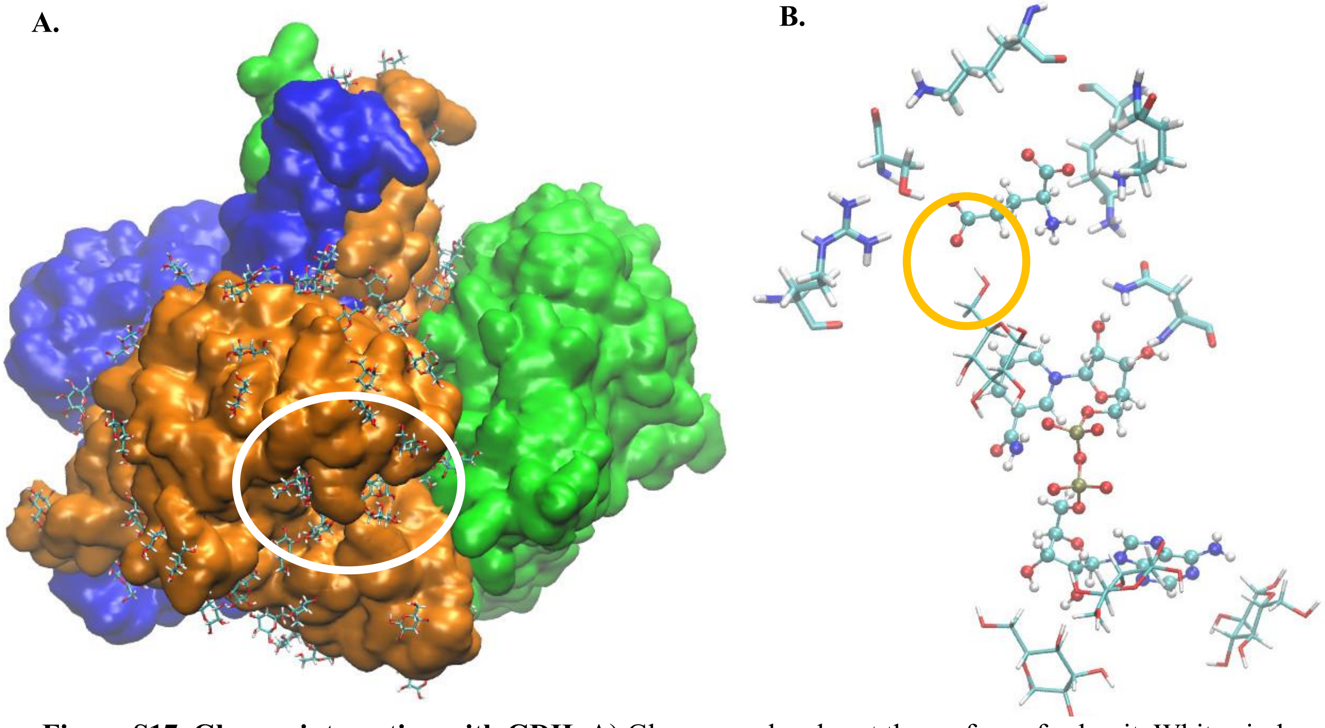

Figure S17. Glucose interaction with GDH. A) Glucose molecules at the surface of subunit. White circle indicates channel to access active site. B) The deepest observed penetration of glucose into the GDH active site. Yellow circle shows H-bond between glutamate and glucose.

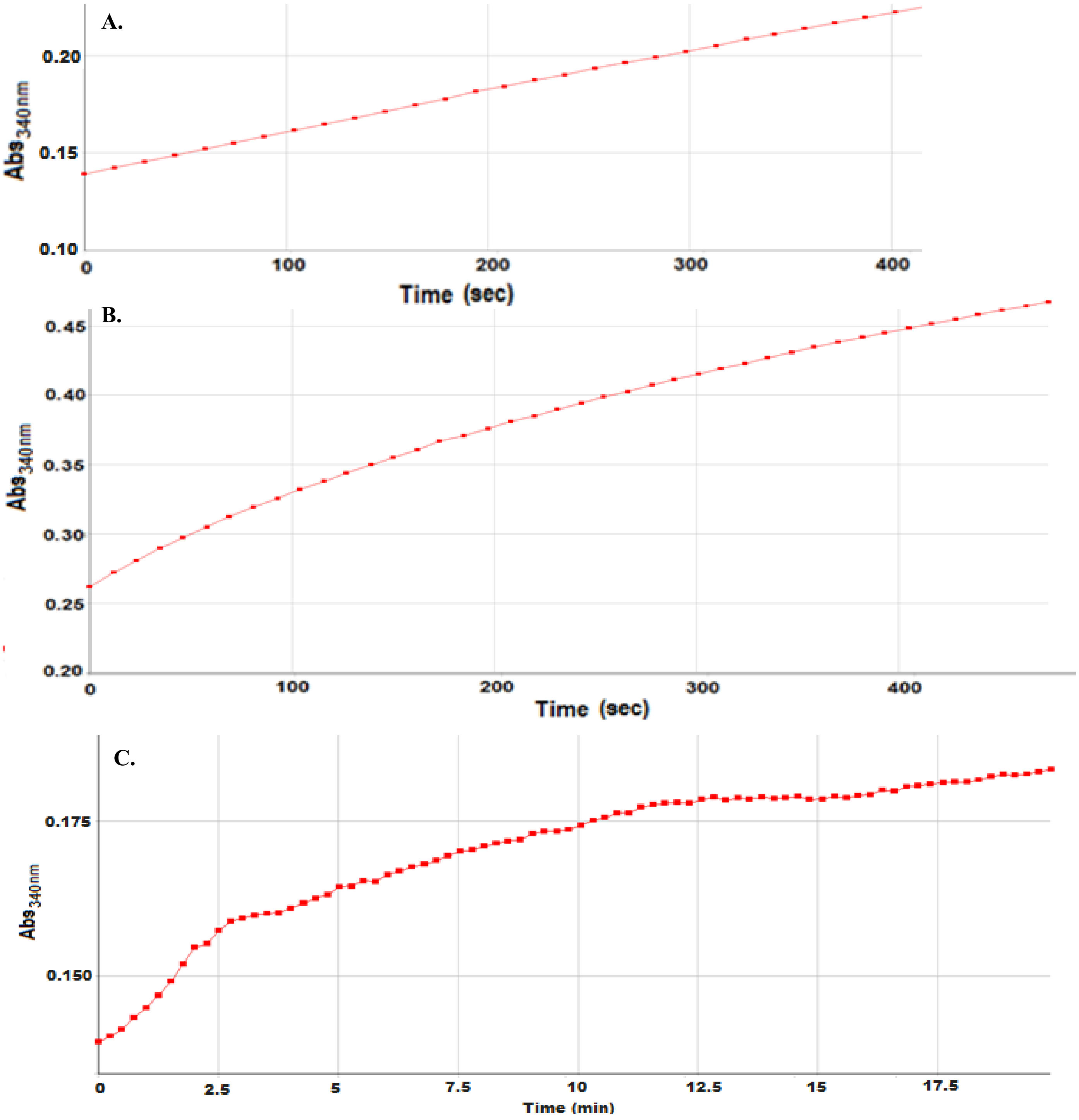

Figure S18. Example absorbance versus time plots for glutamate dehydrogenase assay. Using 9 mM glutamate and 1 mM NAD^+, the absorbance at 340 nm was measured over time for A) 85 nM GDH and B) 160 nM GDH in 100 mM phosphate buffer (pH = 7). C) The resulting curve was less linear when the glutamate concentration was decreased 10-fold using 160 nM GDH.

Table S3. Comparison of crowding effects in 100 mM phosphate vs pyrophosphate buffer at pH 7

	300 g/L Glucose		300 g/L Dextran		300 g/L BSA	
	**Rel V_{max}**	**Rel K_m**	**Rel V_{max}**	**Rel K_m**	**Rel V_{max}**	**Rel K_m**
**Phosphate**	0.4 ± 0.1	0.48 ± 0.06	0.57 ± 0.06	0.6 ± 0.08	0.71 ± 0.07	0.9 ± 0.3
**Pyrophosphate**	0.49 ± 0.02	0.3 ± 0.1	0.7 ± 0.1	0.7 ± 0.1	0.70 ± 0.07	1.0 ± 0.2

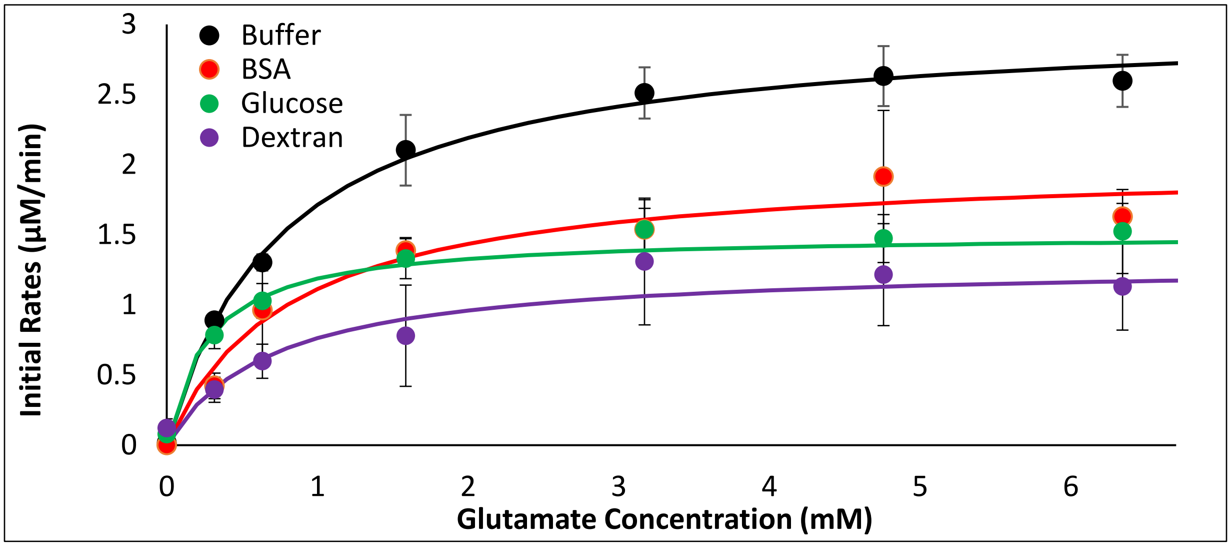

Figure S19. Example Michaelis-Menten curves for glutamate dehydrogenase. Reaction rates were monitored by absorbance at 340 nm while varying glutamate at 1 mM NAD^+ and 120 nM GDH in the presence or absence (black) of 300 g/L bovine serum albumin (BSA, red) glucose (green) or dextran 150 kDa (purple) in 100 mM pyrophosphate buffer at pH 7. Absorbance was converted to µM of NADH product using an extinction coefficient (ε) and pathlength (ℓ) determined from an NADH calibration curve ($\varepsilon\ell$ = 0.0037 μM^{-1}). Error bars represent standard deviations (n = 3).

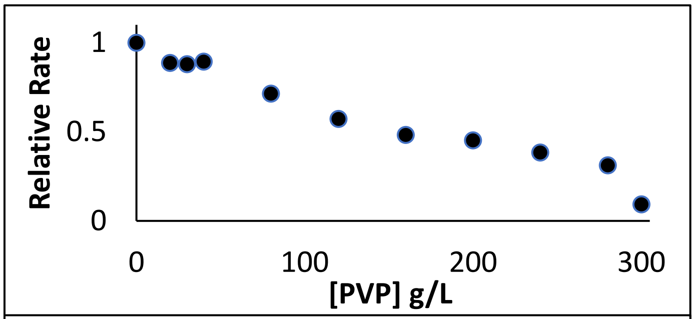

Figure S20. Polyvinylpyrrolidone inhibits GDH activity. At 9 mM glutamate, 1 mM NAD^+, and 60 mM GDH reaction rates were measured in the presence of varying concentrations of 10 kDa PVP in 100 mM phosphate buffer at pH 7.

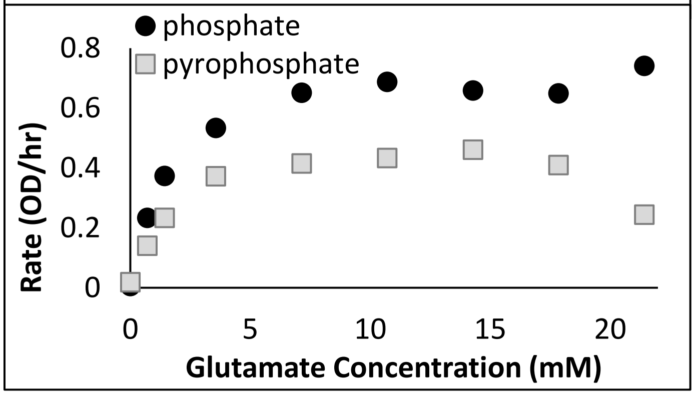

Figure S21. Buffer alters GDH activity. Michaelis-Menten curves were collected at 1 mM NAD^+ and 85 mM GDH in 100 mM phosphate buffer (black) or 100 mM pyrophosphate buffer (grey) at pH 7.

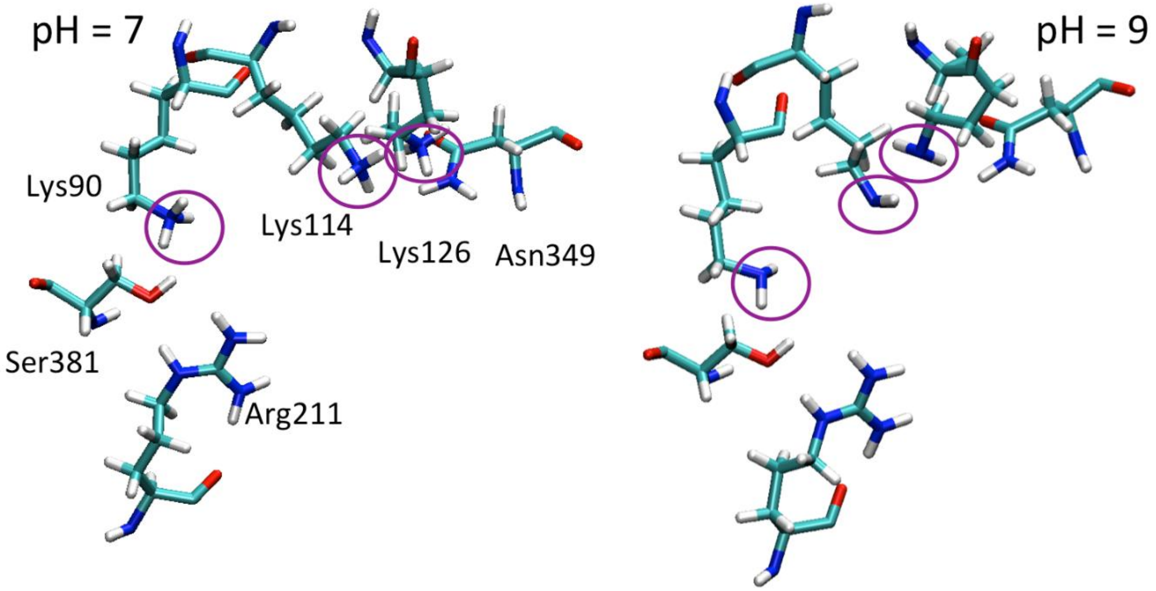

Figure S22. Changes of the structure of the active site caused by the increase of pH. The amino groups of the three Lys residues in purple circles are protonated at pH = 7, but neutral at pH = 9.

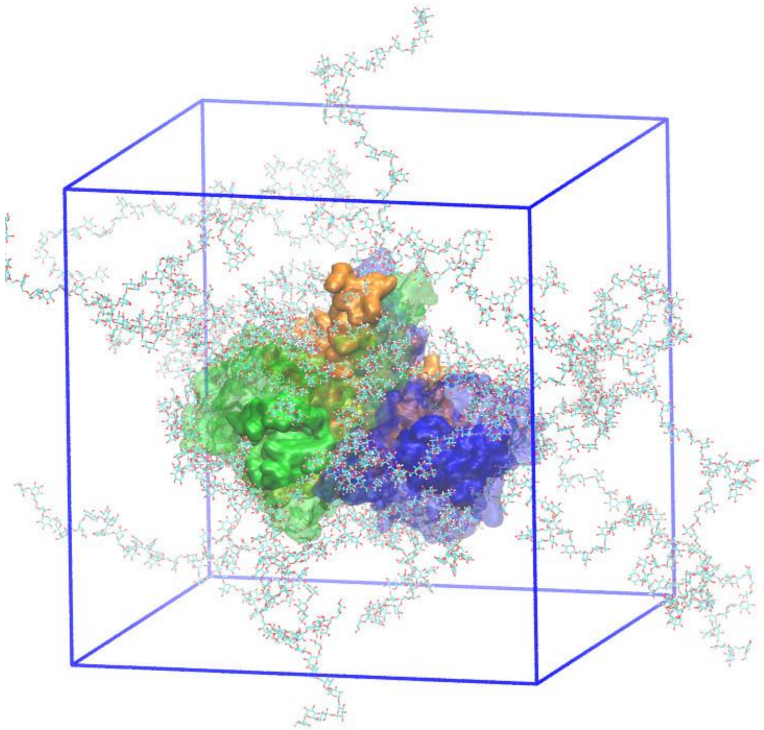

Figure S23. The structure of GDH trimer in crowded environment (dextran) in a cubic simulation cell. All water molecules are omitted for better clarity. Different colors correspond to different subunits: A (blue), B is (orange), C is (green). Dextran crowder is depicted by green-red lines.

Table S4. Composition of the simulated model systems

Substrate	Cofactor and other ligands	Solvent	Crowding agent concentration (g/l)	Number of crowder molecules	pH	Counterions Na^+ (number of atoms)	Average box size (nm)
×	×	H_2O	×	×	7	3	15.223
GLU	NAD^+	H_2O	×	×	7	9	15.224
GLU	NADPH, GTP	H_2O	×	×	7	30	15.223
NVA	NAD^+	H_2O	×	×	7	6	15.224
GLU	NAD^+	H_2O	×	×	9	18	15.222
NVA	NAD^+	H_2O	×	×	9	15	15.221
×	×	glucose in water	100	1172	7	3	15.067
GLU	NAD^+	glucose in water	100	1172	7	9	15.068
GLU	NADPH, GTP	glucose in water	100	1172	7	30	15.066
NVA	NAD^+	glucose in water	100	1172	7	6	15.068
GLU	NAD^+	glucose in water	100	1172	9	18	15.068
NVA	NAD^+	glucose in water	100	1172	9	15	15.067
×	×	dextran in water	100	22	7	3	15.129
GLU	NAD^+	dextran in water	100	22	7	9	15.128
GLU	NADPH, GTP	dextran in water	100	22	7	30	15.124
NVA	NAD^+	dextran in water	100	22	7	6	15.128
GLU	NAD^+	dextran in water	100	22	9	15	15.146
NVA	NAD^+	dextran in water	100	22	9	15	15.146
